# *p53*, *SKP2*, and *DKK3* as MYCN Target Genes and Their Potential Therapeutic Significance

**DOI:** 10.3389/fonc.2012.00173

**Published:** 2012-11-28

**Authors:** Lindi Chen, Deborah A. Tweddle

**Affiliations:** ^1^Newcastle Cancer Centre, Northern Institute for Cancer Research, Newcastle UniversityNewcastle, UK

**Keywords:** neuroblastoma, MYCN, p53, SKP2, DKK3, MDM2-p53 antagonists

## Abstract

Neuroblastoma is the most common extra-cranial solid tumor of childhood. Despite significant advances, it currently still remains one of the most difficult childhood cancers to cure, with less than 40% of patients with high-risk disease being long-term survivors. *MYCN* is a proto-oncogene implicated to be directly involved in neuroblastoma development. Amplification of *MYCN* is associated with rapid tumor progression and poor prognosis. Novel therapeutic strategies which can improve the survival rates whilst reducing the toxicity in these patients are therefore required. Here we discuss genes regulated by MYCN in neuroblastoma, with particular reference to *p53*, *SKP2*, and *DKK3* and strategies that may be employed to target them.

## Introduction

Neuroblastoma, an embryonal malignancy of the developing neural crest, is the most common extra-cranial solid tumor of childhood. It accounts for 8–10% of all pediatric cancers and 15% of childhood cancer mortality (Park et al., 2010). Over 50% of patients present with high-risk metastatic disease at the time of diagnosis and most will respond to intensive multi-modal therapy despite significant acute toxicities. However relapse with chemoresistant disease is common and the overall long-term survival of high-risk patients currently remains less than 40%, with those that survive often having long-term toxicities. Thus there is a continuing need to identify novel, less toxic therapies that will ultimately improve the survival of this subset of patients. A typical feature of high-risk disease is *MYCN* amplification which occurs in ~25% of neuroblastoma, associating with rapid tumor progression and a poor prognosis (reviewed by Cohn and Tweddle, 2004). *MYCN* is a proto-oncogene directly involved in neuroblastoma tumorigenesis, evident by the spontaneous development of neuroblastoma in a MYCN dose-dependent manner in transgenic murine models (Weiss et al., 1997).

Direct inhibition of MYCN has not yet been clinically successful (Gustafson and Weiss, 2010), consequently there is a focus on developing therapeutic strategies directed at destabilizing MYCN protein, and at the downstream targets or pathways which mediate the oncogenic functions of MYCN, and drive the aggressive behavior and progression of *MYCN* amplified tumors. This review will focus on three selected MYCN target genes *p53*, *SKP2*, and *DKK3*, which we have previously identified as being directly or indirectly regulated by MYCN (Bell et al., 2007a; Chen et al., 2010), and strategies that are now being or could be employed in the future to target them, particularly in *MYCN* amplified neuroblastoma.

## MYCN

MYCN belongs to the *MYC* family of basic-helix-loop-helix-leucine zipper (bHLH-LZ) transcription factors which also includes c-MYC, and MYCL. Deregulated expression of *Myc* family members have been implicated in the genesis of several human cancers. Consistent with this, studies have shown that MYC contributes to numerous aspects of tumorigenesis including unrestricted cellular growth and proliferation, angiogenesis, inhibition of differentiation, metastasis, genomic instability, and reduced cell adhesion (reviewed by Adhikary and Eilers, 2005).

The *MYCN* gene located at 2p24 encodes a 64 kDa nuclear phosphoprotein, which contains a transcriptional activation domain at the N-terminal, and a transcriptional regulation domain with a bHLH-LZ motif at the C-terminal (Schwab, 2000). In contrast to c-MYC, which is expressed in a wide variety of embryonic and adult tissues, MYCN expression is limited to the developing nervous system and selected other sites (Cohn and Ikegaki, 2000).

### Transactivation and repression of target genes by MYCN

MYC proteins function as active heterodimers with MAX via their conserved bHLH-LZ domains to exert transcriptional activation via direct binding to E-Box motifs (CANNTG) within target gene promoters and the subsequent recruitment of multiple transcriptional coactivators. Heterodimerization with MAX is required for direct binding of MYC proteins to DNA. *Myc* family members have short half-lives and their expression levels are highly regulated. In contrast, MAX is stable and constitutively expressed, and normally present in stoichiometric excess to MYC, which suggests that the abundance of active heterodimers is dependent on the levels of MYC proteins (reviewed by Grandori et al., 2000).

In contrast to transactivation mediated by MYC, transcriptional repression is independent of E-Box binding and has been shown to involve recruitment of MYC proteins to target gene promoters by Miz-1 and disruption of the interaction between transcriptional complexes. MYC mediated transcriptional repression via Miz-1 has been shown for *p15^INK4B^* (Staller et al., 2001) and *p21^CIP1^* (Seoane et al., 2002). Other candidate proteins which have been proposed to recruit MYC to core promoters include TFII-I, NF-Y, YY-1, and SP1 (reviewed by Wanzel et al., 2003; Adhikary and Eilers, 2005).

### Identifying MYCN target genes

The identification of MYCN target genes enables a greater understanding of MYCN driven neuroblastoma tumorigenesis and promotes the identification of potential targets for therapeutic intervention in the treatment of neuroblastoma. A vast number of c-MYC target genes have been identified and can be found at http://myccancergene.org/site/mycTargetDB.asp (Zeller et al., 2003), however less is known about the target genes of MYCN. It has been estimated that MYC is bound to ~25,000 sites within the human genome (reviewed by Adhikary and Eilers, 2005). Early studies found that several c-MYC target genes were expressed in some neuroblastoma cell lines with *MYCN* amplification, but not all, suggesting that other cell specific factors may be important (Ben-Yosef et al., 1998). More recent studies have reported significant overlap between c-MYC and MYCN-regulated gene sets (Laurenti et al., 2008; Westermann et al., 2008).

Target genes downstream of MYCN can be classified as direct or indirect. Direct target genes of MYCN can be defined as genes which possess a MYCN binding E-Box motif located within close proximity to the transcriptional start site of the gene and/or for which MYCN has been shown to directly bind to the gene promoter to drive transcription. This involves using methods such as electrophoretic mobility shift assay (EMSA), reporter gene assays, and/or more recently Chromatin Immunoprecipitation (ChIP) analysis, a technique which allows specific protein-DNA interactions to be isolated. Indirect target genes of MYCN are genes which are altered as a consequence of other genes or pathways that are directly regulated by MYCN (Bell et al., 2010).

There are several approaches used to identify target genes of transcription factors such as MYCN. The candidate gene approach involves selecting genes which are involved in the known biological functions of MYCN, such as cell proliferation. Furthermore due to the homology between the *Myc* family members, the candidate gene approach is often used to determine whether previously known c-MYC target genes are also MYCN target genes (Bell et al., 2010). Alternatively, the inference approach is used and is based on identifying putative target genes by the presence of MYCN/MAX binding sites within their gene regulatory regions (Dang, 1999). Moreover, target genes may also be identified based on their differential expression in conditions with varying MYCN expression levels, such as MYCN regulatable expression systems or comparing *MYCN* amplified versus non-amplified cell lines and/or tumors. The latter is often performed using microarray based genome-wide approaches (reviewed by Bell et al., 2010).

More recently, with the development of ChIP and advances in technology, genome-wide *in vivo* approaches for identifying protein-DNA interactions have become available, including ChIP-cloning, ChIP-chip, and the latest and increasingly popular, ChIP-seq. These techniques enable the identification of direct target genes, and the specific *in vivo* binding sites within the genome without prior knowledge. Direct targets may be further confirmed using quantitative PCR based ChIP analysis (Wu et al., 2006). Interestingly, a ChIP-chip array study of MYCN/c-MYC target genes in neuroblastoma demonstrated that distinct MYCN/c-MYC target gene expression was associated with overall survival, and independent of well-established markers such as *MYCN* amplification, disease stage, and age at diagnosis (Westermann et al., 2008).

## p53

p53 was discovered over three decades ago as one of the first tumor suppressors (Lane and Crawford, 1979; Linzer and Levine, 1979) and has since been shown to be the most frequently mutated gene in human cancer. p53 is involved in the regulation of several processes that contribute to its central role in maintaining genomic stability and tumor suppression, including cell cycle arrest, apoptosis, senescence, differentiation, autophagy, DNA repair, angiogenesis, cell migration, metabolism, and the immune response. The *TP53* gene located at chromosome position 17p13.1 encodes a 53 kDa nuclear phosphoprotein that consists of an N-terminal transactivation domain (TAD), a central sequence-specific DNA binding domain (DBD), a tetramerization domain, and a highly basic C-terminal regulatory domain. Nuclear export signals (NES) are located within both the N- and C-termini, and three lysine-rich nuclear localization signals (NLS) are located within the C-terminal (reviewed by Bai and Zhu, 2006). Identified more recently, p63 and p73 are two homologs which share structural and functional similarity to p53 and belong to the p53 family (reviewed by Levrero et al., 2000).

### The p53/MDM2/p14^ARF^ pathway

**Figure 1:**
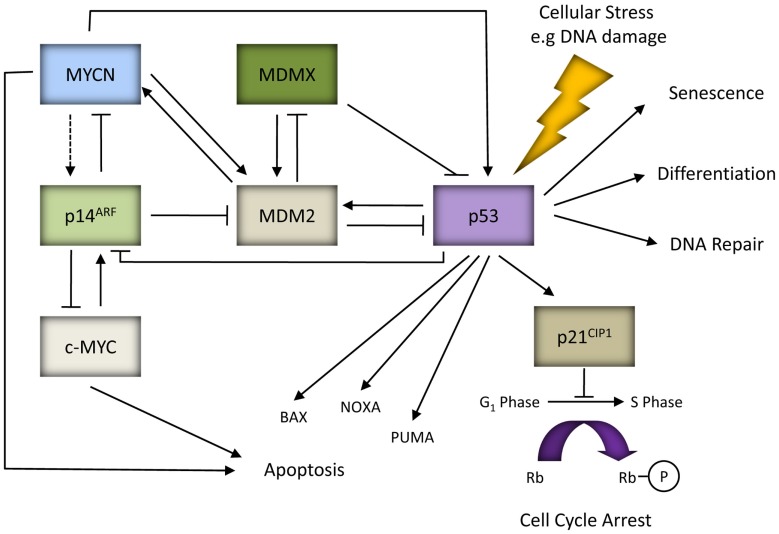
**MYC proteins and the p53/MDM2/p14^ARF^ pathway**. In response to cellular stresses p53 can mediate the expression of genes involved in various cellular responses such as apoptosis (e.g., BAX, NOXA, and PUMA), cell cycle arrest (e.g., p21^CIP1^), differentiation, DNA repair or senescence. p53, MDM2 and p14^ARF^ form an autoregulatory feedback loop to tightly regulate p53 expression and activity. p14^ARF^ can be activated in response to aberrant oncogenic factors such as c-MYC, and possibly MYCN (as indicated by the dashed line). p14^ARF^ can also exhibit p53-independent tumor suppressor activity by directly binding and inhibiting the activity of c-MYC and MYCN. Both *p53* and *MDM2* are direct target genes of MYCN. MDM2 can regulate *MYCN* mRNA stability and translation, thereby forming a positive feedback loop.

Under normal cellular conditions, p53 is maintained at low levels mainly due to MDM2, an E3 ubiquitin ligase and the critical negative regulator of p53 (Honda et al., 1997). This is supported by the observed embryonic lethality of *MDM2* knockout mice and their rescue by the concomitant deletion of *p53* (Jones et al., 1995; Montes de Oca Luna et al., 1995). *MDM2* is a direct transcriptional target of p53 and is induced in response to p53 activation thereby forming a tightly regulated negative feedback loop. MDM2 directly binds to the N-terminal TAD of p53 to inhibit p53 transcriptional activity (Momand et al., 1992), as well as promoting nuclear export and targeting p53 for ubiquitin mediated proteasome degradation (Honda et al., 1997; Tao and Levine, 1999a). p14^ARF^ is a tumor suppressor and the negative regulator of MDM2. Studies have shown that p14^ARF^ promotes p53 stability and activity by inhibiting MDM2-mediated degradation of p53 via direct interaction with MDM2 and inhibiting its E3 ligase activity (Honda and Yasuda, 1999), preventing MDM2 and p53 nuclear export (Tao and Levine, 1999b), sequestering MDM2 within the nucleolus (Weber et al., 1999), and also by promoting MDM2 degradation (Zhang et al., 1998). Activated p53 can subsequently downregulate the expression of *p14^ARF^* (Robertson and Jones, 1998; Stott et al., 1998). MDMX is a homolog of MDM2 and a negative regulator of p53. It has been shown to enhance MDM2-mediated ubiquitination and degradation of p53, and repress p53-mediated transcription (reviewed by Marine et al., 2007; Kruse and Gu, 2009). Interestingly, MDM2 can promote ubiquitination and degradation of MDMX, an effect which is stimulated by p14^ARF^ and which correlates with the ability of p14^ARF^ to bind MDM2 (Pan and Chen, 2003; Figure 1).

### Activation and function of p53

In response to a variety of intrinsic and extrinsic cellular stresses such as DNA damage, oncogenic activation, deprivation of growth factors/signals, ribonucleotide depletion, microtubule disruption, and hypoxia, there is stabilization, nuclear accumulation, and activation of p53. This is predominantly regulated by post-translational modifications conferred on the p53 protein, such as phosphorylation, ubiquitylation, acetylation, and sumoylation (Bode and Dong, 2004). N-terminal modifications of p53 act to inhibit the p53-MDM2 interaction thereby preventing MDM2-mediated inactivation and degradation of p53. In addition, modifications within the DBD and C-terminal of p53 have been shown to increase p53 sequence-specific DNA binding and relieve the inhibitory effect of the C-terminal regulatory domain on the core DBD of p53, respectively (reviewed by Appella and Anderson, 2001). Once activated, p53 initiates cellular responses via transcriptional regulation of a vast repertoire of downstream target genes encoding proteins and microRNAs (miRNAs), as well as transcriptional independent functions. The type of response can be dependent on several factors that are both extrinsic and intrinsic to the cell, such as cell type, cellular environment, oncogenic lesions present in the cell, and also stress type (Jimenez et al., 1999; Vousden and Lu, 2002).

p53 functions as an active tetramer to directly bind to a consensus p53 DNA binding sequence consisting of two adjacent 10 bp half-sites 5′-RRRCWWGYYY-3′ (*R* = A or G, *Y* = C or T, *W* = A or T), separated by 0–13 bp, located within the promoters of downstream target genes (el-Deiry et al., 1992). In contrast, most genes which are repressed by p53 tend to lack p53 DNA binding sites within their promoters (Mirza et al., 2003). To date several mechanisms for p53-mediated transcriptional repression have been identified (reviewed by Wang et al., 2010a; Bohlig and Rother, 2011). The total number of p53 target genes is unknown however it has been shown that 4852 genes within the human genome contain at least one consensus p53 DNA binding site. Furthermore, the identification of non-consensus p53 binding sites also contributes to the ever expanding repertoire of p53 target genes (reviewed by Menendez et al., 2009). p53 target genes have been previously reviewed (Mirza et al., 2003; Harms et al., 2004; Nakamura, 2004; Riley et al., 2008; Wang et al., 2010a).

In recent years, p53 has also been shown to regulate the transcriptional expression and maturation of miRNAs, a class of endogenously expressed small (~18–25 nt) non-coding RNA molecules involved in post-transcriptional regulation of gene expression (Lujambio and Lowe, 2012). p53 has been found to upregulate the expression of the *miR-34* cluster which is reported to mediate several tumor suppressive functions of p53 including senescence, cell cycle arrest, and apoptosis (Bommer et al., 2007; Chang et al., 2007; He et al., 2007; Raver-Shapira et al., 2007; Tarasov et al., 2007). Consistent with this, reduced levels of *miR-34* have been observed in both tumors and cell lines, including neuroblastoma (Bommer et al., 2007; Chang et al., 2007; Tarasov et al., 2007; Welch et al., 2007; Feinberg-Gorenshtein et al., 2009). Additionally, p53 has also been reported to induce the expression of *miR-192*, *miR-215*, *miR-145*, and *miR-107*, of which *miR-145* was shown to inhibit c-MYC expression (Braun et al., 2008; Georges et al., 2008; Sachdeva et al., 2009; Yamakuchi et al., 2010). Conversely, several miRNAs have been found to regulate the abundance and activity of p53, such as *miR-380-5p*, which is associated with poor outcome in *MYCN* amplified neuroblastoma (Swarbrick et al., 2010).

p53 is also able to exert functions via protein-protein interactions, and several proteins involved in cell cycle control, DNA repair, gene transcription, and apoptosis have been shown to bind to p53 (Moll et al., 2005; Braithwaite et al., 2006; Speidel et al., 2006). Although p53 is a nuclear protein, a fraction of p53 has been found within the cytoplasm, exhibiting non-nuclear transcriptional independent activities. Cytoplasmic p53 has been reported to directly interact with members of the BCL2 family including anti-apoptotic BCLxL and BCL2, and proapoptotic BAX and BAK which result in mitochondria membrane permeability, release of cytochrome C, and apoptosis (reviewed by Moll et al., 2005).

### p53 pathway inactivation and cancer

The crucial role p53 plays in tumor suppression is emphasized by observations that the p53 pathway is abrogated in around half of all cancers due to an inactivating *p53* mutation, and the rest have impaired upstream or downstream p53 pathways (Brown et al., 2009). This is further supported by the severe predisposition to cancers observed in individuals with Li-Fraumeni syndrome (Evans and Lozano, 1997). Additionally, *p53* null mice are reported to develop a range of spontaneous tumors, most commonly T-cell lymphomas (Donehower, 1996).

The frequency of *p53* mutations varies from 10 to 70% across different cancers types, and are more common in solid tumors compared with hematological malignancies (Calin et al., 1999; Soussi et al., 2000). The majority are missense mutations resulting in single amino acid substitutions and map within the DBD of p53 (Brosh and Rotter, 2009). In tumors and cell lines, mutational inactivation predominantly leads to the accumulation of high levels of mutant p53 protein. Some p53 mutants have been shown to exert a dominant negative effect on wild-type (wt) p53 owing to the requirement for p53 to function as an active tetramer (Vousden and Lu, 2002). *p53* mutations identified to date can be found within publically available p53 databases such as The International Agency for Research on Cancer (IARC) *TP53* Mutation Database^1^ and The p53 Website^2^. In addition to loss of tumor suppressive function and exerting dominant negative effects against wt p53, it has become increasingly evident that p53 mutants can exhibit new functions independent of wt p53 and can vary depending on the mutation. These new gain-of-function mutants have been reported to play a role in promoting tumorigenesis including increased metastasis and genomic instability, and resistance to anti-cancer therapies (reviewed by Xu, 2008; Brosh and Rotter, 2009; Oren and Rotter, 2010; Hanel and Moll, 2012). Studies using murine models have observed that mutant *p53* knock-in mice develop more aggressive and metastatic tumors in comparison to *p53* null mice, and that the different p53 mutants are associated with distinct tumor patterns (Lang et al., 2004; Olive et al., 2004; Lozano, 2007; Song et al., 2007; Oren and Rotter, 2010).

The prognostic and predictive significance of *p53* somatic mutations in human cancer have been extensively evaluated over the past 15 years however due to inconsistencies have failed to reach clinical practice to guide treatment. This has predominantly been attributed to the use of immunohistochemistry to detect p53 accumulation as a marker of *p53* mutational inactivation which is inaccurate as this detects both wt and mutant p53 in most cases. More recent studies which have used gene sequencing to determine *p53* status have obtained more consistent results. There is a general trend associating *p53* mutations with a poor prognosis and resistance to chemo- and radiotherapy. This has been shown for malignancies of the breast, head and neck, colorectum, and hematopoietic system (Brosh and Rotter, 2009; Olivier and Taniere, 2011).

In addition to *p53* mutations, amplification, and/or overexpression of *MDM2* or *MDMX*, as well as *p14^ARF^* mutation, deletion, or methylation can also lead to abrogation of the p53 pathway (reviewed by Brown et al., 2009). Similar to p53 null mice, mice deficient in *p14^ARF^*, or overexpressing *MDM2* or *MDMX* also developed spontaneous tumors, albeit at a slower rate (Jones et al., 1998; Kamijo et al., 1999; Xiong et al., 2010). Abrogation of p53 function may also occur as the result of viral inactivation such as the human papillomavirus, or impaired upstream signaling and/or downstream mediators of p53 function, such as inactivation of *ATM* or *CHK2*, or genes involved in the apoptotic response, and/or cell cycle arrest. Although it is not clear why certain mechanisms of p53 pathway inactivation are favored in some tumors but not others, it is likely to be influenced by the various selective pressures acting upon the cancer (Junttila and Evan, 2009).

Interestingly in contrast to *p53*, *p63*, and *p73* are rarely mutated in human cancers and neither *p63* nor *p73* knockout mice exhibit an increased susceptibility to developing spontaneous tumors (Moll and Slade, 2004).

### p53 and neuroblastoma

#### p53 accumulation and function in neuroblastoma

In contrast to many other human cancers, *p53* mutations in neuroblastoma are rare, occurring in ~3% of cases analyzed to date (Imamura et al., 1993; Komuro et al., 1993; Ohgaki et al., 1993; Vogan et al., 1993; Castresana et al., 1994; Hosoi et al., 1994; Kusafuka et al., 1997; Manhani et al., 1997; Omura-Minamisawa et al., 2001; Tweddle et al., 2001b; Carr-Wilkinson et al., 2010). Despite this, p53 has been reported to accumulate in neuroblastoma, which has been suggested to be due to the embryonic nature of these tumors, reflecting a failure of precursor cells to mature (Sidell and Koeffler, 1988; Davidoff et al., 1992). This is consistent with studies which have shown a decrease in p53 expression following retinoic acid induced *in vitro* differentiation of neuroblastoma cell lines (Sidell and Koeffler, 1988; Davidoff et al., 1992; Chen et al., 2007), and also during neuronal development/differentiation (Eizenberg et al., 1996; Ferreira and Kosik, 1996). In addition siRNA mediated inhibition of p53 in neuroblastoma cell lines led to morphological evidence of differentiation (Carr-Wilkinson et al., 2011). The presence of high levels of accumulated wt p53 suggests that neuroblastoma circumvent the tumor suppressive properties of p53 by a mechanism independent of mutation. Early studies reported cytoplasmic sequestration of wt p53 as a non-mutational mechanism for p53 inactivation and accumulation in neuroblastoma (Moll et al., 1995, 1996). To date several mechanisms for cytoplasmic sequestration of p53 in neuroblastoma have been proposed, including masking of the p53 C-terminal nuclear localization signal (Ostermeyer et al., 1996), hyperactive nuclear export (Stommel et al., 1999), binding to the cytoplasmic anchor, Parc (Nikolaev and Gu, 2003; Nikolaev et al., 2003), aberrant hyperubiquitylation of p53 (Becker et al., 2007), and MDMX and MDM2-mediated cytoplasmic tethering (Ohtsubo et al., 2009). In contrast, a number of studies including our own have reported predominantly nuclear localization and/or functional p53 in neuroblastoma (Layfield et al., 1995; Hoehner et al., 1997; Danks et al., 1998; Isaacs et al., 1998; McKenzie et al., 1999; Smart et al., 1999; Keshelava et al., 2000, 2001; Tweddle et al., 2001a,b; Cui et al., 2002; Goldschneider et al., 2004; Chen et al., 2007, 2010; Xue et al., 2007; Kurata et al., 2008; Van Maerken et al., 2011; Gamble et al., 2012). The conflicting results over the localization and function of p53 are likely to be due to the different p53 antibodies and the criteria used to define functional p53. Interestingly, despite functional, transcriptionally active p53, we have previously reported a failure for *MYCN* amplified neuroblastoma cells to G_1_ arrest after DNA damage (Tweddle et al., 2001b; Bell et al., 2006) and have recently shown that the outcome of the p53-mediated response to DNA damage in neuroblastoma cell lines is determined by both morphological subtype (neuronal or substrate adherent) and MYCN expression (Carr-Wilkinson et al., 2011).

#### Inactivation of the p53 pathway and resistance to cytotoxic therapy

Most neuroblastomas initially respond well to cytotoxic agents however the disease frequently relapses with acquired resistance to therapy thus posing a major problem in the treatment of patients with high-risk neuroblastoma. Consequently, gaining a greater understanding into the mechanisms behind resistance to cytotoxic therapies could lead to the development and use of novel therapies in progressing or relapsed disease (Tweddle et al., 2001a). An important mechanism for chemo- and radioresistance is inactivation of the p53 pathway (reviewed by Levine, 1997). The initial good response to chemotherapy may be partly due to the presence of functional p53 at diagnosis, and the development of resistance at relapse may be the result of acquired p53 inactivation at a later stage (reviewed by Tweddle et al., 2003). Consistent with this, of the few *p53* mutations which have been identified in neuroblastoma to date, the majority were in tumors from patients with progressive or relapsed disease and/or post-chemotherapy (Imamura et al., 1993; Komuro et al., 1993; Ohgaki et al., 1993; Vogan et al., 1993; Castresana et al., 1994; Hosoi et al., 1994; Kusafuka et al., 1997; Manhani et al., 1997; Omura-Minamisawa et al., 2001; Tweddle et al., 2001b; Carr-Wilkinson et al., 2010). Similarly, despite the frequency being slightly higher than those in tumors, *p53* mutations in neuroblastoma cell lines are also rare, and the majority were also identified in cell lines established at relapse (Tweddle et al., 2003; Carr et al., 2006; Table 1).

**Table 1:** ***MYCN* status and aberrations in the p53/MDM2/p14^ARF^ pathway in neuroblastoma cell lines**.

Cell Line	*MYCN* Status	*p53* Mut	*p53* Mut Codon	*MDM2* Amp	*p14^ARF^* Deln	*p14^ARF^* Meth	Diagnosis/ Relapse^§^	Reference
ACN	nMNA	Yes	113				Relapse	Forbes et al. (2008)
CHLA-119	MNA^1^	Yes	342				Relapse	Keshelava et al. (2001)
CHLA-172	nMNA^1^	Yes	216				Relapse	Keshelava et al. (2001)
CHLA-90	nMNA	Yes	286				Relapse	Keshelava et al. (2001)
CLB-Pe	MNA	Yes	176				Relapse	Mergui et al. (2008)
IGRN91	MNA	Yes	^a^				Relapse	Goldschneider et al. (2004, 2006)
IGR-NB8	MNA	Yes	326				Diagnosis	Goldschneider et al. (2006)
KELLY/N-206	MNA	Yes	177				Unknown	Mergui et al. (2008), Van Maerken et al. (2011)
KP-N-YS	MNA	Yes	135				Diagnosis^2^	Forbes et al. (2008)
LAN-1	MNA	Yes	182				Relapse	Davidoff et al. (1992), Van Maerken et al. (2011)
LAN-2	MNA	Yes	337				Diagnosis	Van Maerken et al. (2011)
NB4	MNA	Yes	173				Diagnosis^3^	Teitz et al. (2002)
NB6	MNA	Yes	282				Relapse	Teitz et al. (2002)
NB8	MNA	Yes	^b^				Diagnosis	Teitz et al. (2002), Van Maerken et al. (2011)
NB12	MNA	Yes	173				Unknown	Teitz et al. (2002)
NB13	MNA	Yes	173				Unknown	Teitz et al. (2002)
NB14	MNA	Yes	^c^				Diagnosis	Teitz et al. (2002)
NB15	MNA	Yes	248				Unknown	Teitz et al. (2002)
NB20	MNA	Yes	248				Unknown	Teitz et al. (2002)
NLF	MNA	Yes	203				Diagnosis^4^	Van Maerken et al. (2011)
NMB	MNA	Yes	245				Relapse	Goldman et al. (1996)
SJNB-4	MNA	Yes	176				Relapse	McPake et al. (1998)
SKNAS	nMNA	Yes	^d^				Relapse	Goldschneider et al. (2006), Nakamura et al. (2007), Van Maerken et al. (2011)
SKNBE2C	MNA	Yes	135				Relapse	Kaghad et al. (1997), Keshelava et al. (2001), Tweddle et al. (2001a,b) Van Maerken et al. (2011)
SK-N-DZ	MNA	Yes	110				Relapse^5^	Forbes et al. (2008)
SK-N-FI	nMNA	Yes	246				Relapse	Van Maerken et al. (2006, 2011)
TGW	MNA	Yes	282				Relapse	Sugiyama et al. (2003)
NGP	MNA			Yes			Relapse	Corvi et al. (1995), Tweddle et al. (2001b), Van Maerken et al. (2011)
NB1691	MNA			Yes			Relapse	McKenzie et al. (1999)
CHLA134	MNA			Yes			Relapse	Keshelava et al. (2001)
LS	MNA			Yes			Relapse	Corvi et al. (1995), Carr et al. (2006)
TR-14	MNA			Yes			Relapse	Carr et al. (2006), Van Maerken et al. (2011)
LAN-6	nMNA				Yes		Relapse	Keshelava et al. (2001), Thompson et al. (2001), Van Maerken et al. (2011)
SHEP	nMNA				Yes		Relapse	Tweddle et al. (2001b), Carr et al. (2006), Van Maerken et al. (2011)
CHLA-101	MNA				Yes ^e^		Relapse	Thompson et al. (2001)
CHLA-174	nMNA				Yes		Relapse	Thompson et al. (2001)
CHLA-179	MNA				Yes		Relapse	Thompson et al. (2001)
STA-NB-3	MNA				Yes		Relapse^6^	Van Maerken et al. (2011)
GIMEN	nMNA					Yes	Relapse	Carr et al. (2006)
PER-108	MNA					Yes	Relapse	Carr et al. (2006)

*Mut, mutation; Amp, amplification; Deln, deletion; Meth, methylation; MNA, *MYCN* amplified; nMNA, non-*MYCN* amplified*.

*^§^Any cell line established prior to chemo/radiotherapy was classified as established at diagnosis and any cell lines established post-treatment was classified as established at relapse*.

*^1^Personal communication with P. Reynolds, ^2^Personal communication with T. Sugimoto, ^3^Personal communication with S. Ragsdale, ^4^Personal communication with G. Brodeur, ^5^Personal communication with L. Helson, ^6^Personal communication with P. Ambros*.

*^a^Duplication of exons 7–9 between exons 9 and 10 resulting in an extra ~321nts, ^b^Deletion of codons 105-125, ^c^Deletion of codons 126–132, ^d^Deletion of exons 10 and 11 resulting in truncated product ~369nts, ^e^104 bp deletion*.

Using a pair of neuroblastoma cell lines derived from the same patient, before and after cytotoxic therapy, we previously demonstrated that the cell line derived after therapy, at disease relapse, had mutant non-functional p53, and was more resistant to chemotherapy compared to the cell line derived at diagnosis before therapy, which had wt functional p53 (Tweddle et al., 2001a). Other studies have also correlated loss of p53 function with drug resistance in neuroblastoma cell lines, and found that transfection of wt p53 drug sensitive cell lines with E6 vectors led to chemoresistance (Keshelava et al., 2000, 2001). In line with these observations, we recently reported that the presence of a *p53* mutation was independently prognostic for overall survival in neuroblastoma patients (Carr-Wilkinson et al., 2010).

In addition to *p53* mutations, *MDM2* amplification, and *p14^ARF^* deletion or methylation have also been reported in neuroblastoma tumors and cell lines, most of which were from patients with progressive or relapsed disease and/or post-chemotherapy (Corvi et al., 1995; Omura-Minamisawa et al., 2001; Thompson et al., 2001; Gonzalez-Gomez et al., 2003; Su et al., 2004; Carr et al., 2006; Spitz et al., 2006; Caren et al., 2008; Carr-Wilkinson et al., 2010; Wolf et al., 2010). Interestingly, in our recent study of paired neuroblastoma tumors established at diagnosis and relapse which demonstrated a high frequency of p53/MDM2/p14^ARF^ pathway abnormalities in relapsed neuroblastoma, a higher frequency of abnormalities involving *MDM2* amplification, and *p14^ARF^* inactivation (35%) compared to *p53* mutations (15%) was observed. In all three cases with *MDM2* amplification and 8/12 cases with a *p14^ARF^* alteration, the abnormality was present in both the diagnostic and relapsed specimen, whereas in 5/6 cases where a *p53* mutation was detected it was present in the relapsed specimen alone. This therefore suggests that for *MDM2* amplification and *p14^ARF^* alterations their presence predisposes to relapse whereas *p53* mutations are acquired at relapse (Carr-Wilkinson et al., 2010).

In recent years there has been increased interest in assessing whether polymorphisms can influence cancer risk and clinical outcome. A single-nucleotide polymorphism (SNP) in the *MDM2* promoter (SNP309_T to G_) leading to high levels of *MDM2* expression has been found in some tumors and is associated with a poor prognostic outcome, including neuroblastoma (Cattelani et al., 2008; Perfumo et al., 2008). In *p53*, a SNP at codon 72 which leads to an Arg > Pro substitution has previously been identified, and the Arg72 variant has been shown to exhibit enhanced apoptotic capability compared with the Pro72 variant (Dumont et al., 2003). Very recently, analysis of the *p53* codon 72 Arg/Pro polymorphism identified the Pro/Pro phenotype as an independent marker of poor prognosis in neuroblastoma patients, and *in vitro* led to reduced levels of apoptosis in response to chemotherapy and irradiation (Cattelani et al., 2012).

#### MYCN mediated upregulation of p53 as a mechanism for MYCN induced apoptosis in neuroblastoma

Members of the *Myc* family are known to play a paradoxical role in driving both cellular proliferation and inducing apoptosis, which is thought to provide a safeguard mechanism to prevent uncontrolled cellular proliferation and oncogenic transformation. This paradox is observed histologically by a high mitosis-karyorrhexis index, a combined index of both proliferation and apoptosis, in both human *MYCN* amplified neuroblastoma tumors (Shimada et al., 1995, 1999; Goto et al., 2001; Altungoz et al., 2007) and TH*-MYCN* transgenic mouse neuroblastoma tumors (Moore et al., 2008).

*p53* has long been known to be a direct target gene of c-MYC, and mediate c-MYC induced apoptosis (Reisman et al., 1993; Hermeking and Eick, 1994; Roy et al., 1994; Zeller et al., 2003). Initial studies in neuroblastoma showed that *MYCN* amplified tumors expressed significantly higher levels of *p53* mRNA in comparison with non-amplified tumors (Raschella et al., 1991; Berwanger et al., 2002; Westermann et al., 2008), and higher p53 protein expression in the presence of ectopic MYCN in cell lines (Cui et al., 2005; Bell et al., 2006; Sugihara et al., 2006). Using ChIP-chip arrays MYCN was reported to bind to an E-Box within the *p53* promoter, however this study did not include the functional upregulation of *p53* (Westermann et al., 2008). Most recently, in a study of p53 expression, accumulation, and function in neuroblastoma in relation to *MYCN* amplification and expression, we demonstrated that p53 protein expression correlated with MYCN protein expression in both neuroblastoma tumors and cell lines (Chen et al., 2010). Furthermore, we demonstrated that p53 was functional and exhibited greater transcriptional activity in the presence of MYCN leading to the increased expression of several p53 target genes. Finally, we showed that MYCN mediates transactivation of *p53* by interacting directly with the same E-Box motif (CATGTG) as previously reported for c-MYC (Reisman et al., 1993; Figure 1), and is likely to be an important and direct mechanism by which MYCN is able to sensitize cells to p53-mediated apoptosis (Chen et al., 2010). This is consistent with, and may help to explain why human *MYCN* amplified and TH*-MYCN* transgenic mouse neuroblastoma tumors have high levels of apoptosis, and *MYCN* amplified and Tet21N MYCN+ neuroblastoma cells undergo higher levels of apoptosis in response to chemotherapeutic agents (Fulda et al., 1999, 2000; Paffhausen et al., 2007; Chesler et al., 2008), irradiation (Bell et al., 2006), and MDM2-p53 antagonists (Gamble et al., 2012). Certainly, siRNA mediated inhibition of p53 led to reduced levels of apoptosis in *MYCN* amplified neuroblastoma cell lines (Chesler et al., 2008; Chen et al., 2010).

Further support for MYCN driven p53-dependent apoptosis being an important mechanism for tumor suppression in neuroblastoma comes from *in vivo* work using *p53* or *Mdm2* haploinsufficient models of neuroblastoma. MYCN driven tumor formation had higher penetrance and reduced latency in *p53* haploinsufficient mice, and chemotherapy induced apoptosis was shown to be p53-dependent, in which apoptosis was significantly reduced in TH-*MYCN* *p53* +/- tumors compared with TH-*MYCN* *p53* + / + tumors (Chesler et al., 2008). In contrast, *Mdm2* deficiency suppressed MYCN driven neuroblastoma tumorigenesis, as evident by an extended tumor latency and survival and reduced tumor incidence and growth in TH-*MYCN* *Mdm2* +/− transgenic mice compared with TH-*MYCN* *Mdm2* + / + mice. Additionally, TH-*MYCN* *Mdm2* +/− tumors commonly exhibited methylation of *p19*^*ARF*^. These observations thereby demonstrate the necessity of MYCN to overcome p53-mediated tumor suppression during neuroblastoma tumorigenesis either via direct inhibition of p53 by MDM2 or suppression of the p14^ARF^/p53 pathway (Chen et al., 2009).

The ability of MYCN to induce apoptosis, such as through direct upregulation of p53, may be a potential pathway for spontaneous regression. Certainly, a study analyzing MYCN/c-MYC target gene expression and outcome in neuroblastoma has previously suggested p53 as a strong candidate involved in spontaneous regression of 4s tumors (Westermann et al., 2008).

MYCN and p53 are both expressed in the normal embryonic developing nervous system, during the phase of precursor cell expansion prior to the onset of differentiation. In the context of normal embryonic development, direct upregulation of *p53* by MYCN may be an important mechanism to eliminate any rapidly proliferating neuroblasts exposed to potential teratogens, to prevent deregulated proliferation and aberrations during development.

**Figure 2:**
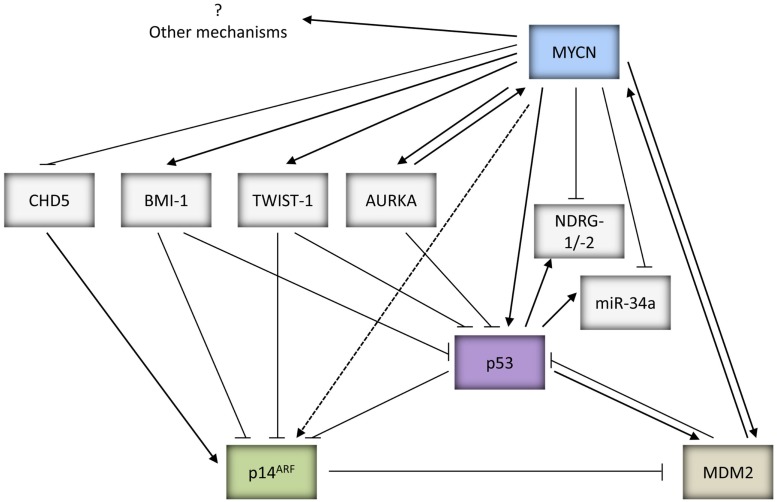
**Summary of the potential mechanisms by which MYCN can both positively and negatively regulate p53 activity and function**.

In addition to direct transcriptional upregulation of *p53*, c-MYC has been previously reported to sensitize cells to increased apoptosis via induction of p14^ARF^ mediated upregulation of p53 expression, stability, and activity (Zindy et al., 1998). Due to the homology between c-MYC and MYCN, MYCN may also be able to stabilize p53 via p14^ARF^, however this remains to be proven experimentally (Figures 1 and 2).

#### Mechanisms of p53 inactivation and evasion of p53-dependent apoptosis in *MYCN* amplified neuroblastoma

Several lines of evidence from published literature lend support to the notion that during the process of neuroblastoma progression there is evasion of p53-mediated tumor suppression via inactivation of the p53/MDM2/p14^ARF^ pathway (reviewed by Van Maerken et al., 2009b) as well as a requirement for *MYCN* amplified neuroblastoma to circumvent MYCN driven apoptosis (reviewed by Hogarty, 2003). *In vivo* studies have demonstrated the importance of the p53 pathway in neuroblastoma (Chesler et al., 2008; Chen et al., 2009). Potential mechanisms whereby *MYCN* amplified tumors can overcome p53-mediated tumor suppression in neuroblastoma are discussed below.

#### *MYCN* amplification and inactivation of the p53/MDM2/p14^ARF^ pathway in neuroblastoma

There is evidence from neuroblastoma cell line studies that *MYCN* amplified neuroblastoma cells may circumvent MYCN driven p53-dependent apoptosis by selecting for cells with aberrations in the p53/MDM2/p14^ARF^ pathway. Analysis of neuroblastoma cell lines reported to date with aberrations in the p53/MDM2/p14^ARF^ pathway demonstrates that 31/40 (78%) of these cell lines are *MYCN* amplified and predominantly established following previous cytotoxic therapy at relapse (Table 1), when abnormalities of the p53 pathway in neuroblastoma tumors have also been previously reported (reviewed by Tweddle et al., 2003; Carr-Wilkinson et al., 2010). In contrast, the proportion of neuroblastoma tumors with aberrations in the p53/MDM2/p14^ARF^ pathway which are *MYCN* amplified are lower than in cell lines (Corvi et al., 1995; Gonzalez-Gomez et al., 2003; Su et al., 2004; Spitz et al., 2006; Caren et al., 2008; Carr-Wilkinson et al., 2010), therefore it is possible that these abnormalities are selected for during the *in vitro* establishment and/or maintenance of these cell lines. It is however important to note that *MYCN* amplification and/or overexpression is unlikely to be the only selection pressure for p53 inactivation as aberrations in the p53 pathway are also found in non-*MYCN* amplified tumors and cell lines (Table 1; Carr et al., 2006; Carr-Wilkinson et al., 2010).

##### MDM2-mediated MYCN driven tumorigenesis

*MDM2* is a direct target gene of MYCN (Slack et al., 2005; Westermann et al., 2008) and non-syntenic co-amplification of *MDM2* and *MYCN* has been reported in neuroblastoma (Corvi et al., 1995). Consistent with observations that *Mdm2* haploinsufficiency inhibits c-MYC induced lymphomagenesis (Alt et al., 2003), MDM2 has been shown to be necessary for MYCN to overcome p53-mediated tumor suppression for MYCN directed centrosome amplification and genomic instability (Slack et al., 2007), and also during MYCN driven neuroblastoma tumorigenesis (Chen et al., 2009; Figure 2). The importance of MDM2 in neuroblastoma pathogenesis is further illustrated by studies which have observed that a SNP within the *MDM2* promoter (SNP309_T to G_) that can lead to higher expression of MDM2 and greater inhibition of p53, is associated with poor survival in neuroblastoma, in particular stage 4 patients with *MYCN* amplification (Cattelani et al., 2008; Perfumo et al., 2008, 2009).

An additional mechanism whereby MYCN can overcome p53-mediated apoptosis is through reduced expression of *CHD5*, a tumor suppressor gene (TSG) mapping to 1p36.31, which is a region commonly deleted in neuroblastoma and frequently correlated with *MYCN* amplification (Figure 2). CHD5 has been shown to control proliferation, apoptosis, and senescence by inducing *p19^ARF^*, the negative regulator of MDM2, and stabilizing p53 (Bagchi et al., 2007). Low levels of *CHD5* expression have been found in neuroblastoma cell lines, as well as correlating with *MYCN* amplification and poor prognosis in neuroblastoma tumors (Thompson et al., 2003; Fujita et al., 2008; Koyama et al., 2012).

Furthermore, MYCN can also suppress p53 through Aurora Kinase A (AURKA), a serine/threonine kinase which plays a key role in mitosis and is expressed at high levels in *MYCN* amplified neuroblastoma (Figure 2). AURKA has been shown to directly phosphorylate p53 at Ser-215 and Ser-315 which abrogates p53 DNA binding and transactivation of target genes (Liu et al., 2004), and promotes MDM2-mediated destabilization and inhibition of p53 (Katayama et al., 2004). There is additional evidence to suggest that AURKA can also inhibit p53 via the AKT/MDM2 axis in gastric cancer cells, however this remains to be shown in neuroblastoma (Dar et al., 2008). Interestingly, AURKA has also been reported to stabilize MYCN, which would further promote MYCN driven tumorigenesis (Otto et al., 2009; Figure 2).

Very recently, MDM2 was reported to play a p53-independent role by interacting directly with *MYCN* mRNA and regulating its stabilization and translation, thereby forming a positive feedback loop, critical for *MYCN* amplified neuroblastoma tumor cell growth and survival (Gu et al., 2012; Figures 1 and 2).

##### Twist-1 and BMI-1 mediated inhibition of the p53 pathway

Twist-1 which belongs to the bHLH transcription factor family, and BMI-1, a polycomb ring finger oncogene, are both overexpressed in several human cancers, and have been shown to be involved in epithelial-mesenchymal transition and cancer stemness which have clinical implications of cancer metastasis, drug resistance, and survival (Wu et al., 2012a). *Twist-1* expression has been shown to be consistently overexpressed in *MYCN* amplified neuroblastoma tumors and cell lines, correlating with MYCN expression (Valsesia-Wittmann et al., 2004). Studies have demonstrated the ability of Twist-1 to inhibit p53 expression and activity via both transcriptional and post-translational mechanisms (Figure 2). Twist-1 has been shown to directly interact with HOXA5, a potent transactivator of p53 (Stasinopoulos et al., 2005). Additionally, it interferes with p53 stabilization and activity via inhibition of the p53/p14^ARF^ pathway by reducing *p14^ARF^* levels (Maestro et al., 1999), and inhibition of Ser-20 phosphorylation in response to DNA damage (Stasinopoulos et al., 2005). Furthermore, Twist-1 has been reported to repress p53-mediated gene transcription and stability via inhibition of p300-mediated acetylation of p53 (Hamamori et al., 1999). Moreover, it is found to directly interact with the DBD of p53, thereby inhibiting p53-mediated transactivation of downstream target genes (Shiota et al., 2008). Finally, very recently Twist-1 has also been reported to interact with the C-terminal regulatory domain of p53, which hinders post-translational modifications of p53 including Ser-392 phosphorylation, and promotes MDM2-mediated p53 degradation (Piccinin et al., 2012).

*BMI-1* is a direct transcriptional target of c-MYC and MYCN and is overexpressed in ~90% of neuroblastoma, correlating with MYCN expression (Ochiai et al., 2010; Huang et al., 2011b). It is reported to repress the *CDKN2A* (*p16^INK4a^*/*p14^ARF^*) locus which can lead to inactivation of the p53 pathway. In neuroblastoma, BMI-1 is shown to be essential for neuroblastoma tumorigenesis *in vitro* and *in vivo*, cooperating with MYCN via inhibition of MYCN driven apoptosis and downregulation of p53 expression (Cui et al., 2007; Huang et al., 2011b). Very recently, as an additional mechanism for BMI-1 mediated p53 inactivation, BMI-1 was found to directly bind to p53 in a complex with other Polycomb complex proteins in neuroblastoma cells leading to increased p53 ubiquitination and degradation (Calao et al., 2012; Figure 2).

##### Downregulation of NDRG1 and NDRG2 mediated p53-dependent apoptosis

*NDRG1* and *NDRG2* were originally identified as a genes downregulated by MYCN (Shimono et al., 1999; Li and Kretzner, 2003; Zhang et al., 2006, 2008). The promoter of *NDRG1* has been reported to possess a p53 binding site and leads to p53-mediated transactivation. *NDRG1* was shown to be upregulated by p53 after DNA damage, and inhibition of *NDRG1* expression by siRNA abolishes p53-dependent caspase activation and apoptosis. Furthermore, NDRG1 has been found to suppress metastatic cell growth (Stein et al., 2004). Similar results have been reported for NDRG2 (Liu et al., 2008a). Downregulation of *NDRG1* and *NDRG2* may therefore enable *MYCN* amplified and/or overexpressing neuroblastoma to evade p53 driven apoptosis (Figure 2).

##### MYCN and miRNAs

The 3′-UTR of *MYCN* has been identified as a direct target of *miR-34a* (Wei et al., 2008), a miRNA which is directly upregulated by p53 and mediates several tumor suppressive functions of p53 (reviewed by Hermeking, 2007). Studies have shown that *miR-34a* plays a role in inhibiting cellular proliferation and inducing apoptosis in neuroblastoma cells (Welch et al., 2007; Cole et al., 2008). Interestingly, *miR-34a* is located at 1p36.23, a region showing frequent loss of heterozygosity (LOH) in neuroblastoma and which is associated with *MYCN* amplification and aggressive disease progression (reviewed by Wei et al., 2008). Consistent with this, lower expression levels of *miR-34a* have been reported to correlate with 1p36 LOH in neuroblastoma (Welch et al., 2007; Cole et al., 2008; Feinberg-Gorenshtein et al., 2009; Figure 2). More recently, *miR-380-5p*, a miRNA which is reported to repress p53, has been shown to be expressed at high levels in neuroblastoma correlating with poor outcome in *MYCN* amplified tumors. Anti-*miR-380-5p* treatment restored p53 function in p53 wt neuroblastoma cell lines and inhibited the growth of orthotopically transplanted TH-*MYCN* tumors (Swarbrick et al., 2010).

### Therapeutic strategies to overcome mechanisms of p53 inactivation in neuroblastoma

#### MDM2-p53 antagonists

The importance of p53 in human cancer has led to vast efforts in the development of p53-based cancer therapeutics (reviewed by Lane et al., 2010). One such approach is the development of small molecule inhibitors (SMIs) which disrupt the interaction between p53 and its negative regulator MDM2 to reactivate wt p53, as a novel class of anti-cancer agents. This class includes *cis*-imidazolines (e.g., Nutlins), spiro-oxindoles (MI compounds), benzodiazepinediones, isoindolinones, isoquinolinones, and thiophenes (RITA; reviewed by (Yuan et al., 2011); (Hardcastle et al., 2011)). All of these act by targeting the p53 binding pocket of MDM2 to inhibit the MDM2-p53 interaction, except RITA which binds directly to p53. Compounds that bind to the RING domain of MDM2 and act in part by inhibiting the interaction of MDM2 with the proteasome have also been developed and include 5-deazaflavins and tryptamines (reviewed by Yuan et al., 2011).

Nutlins were the first potent and selective inhibitors of the MDM2-p53 interaction (Vassilev et al., 2004), in particular Nutlin-3 has been extensively evaluated *in vitro* and *in vivo* in several types of human cancers and the *cis*-imidazoline RG7112 is currently in phase I clinical trials^3^ (NCT00559533 and NCT00623870). Overall, MDM2-p53 antagonists have been shown to activate the p53 pathway, inducing p53-dependent apoptosis and sensitizing tumor cells to cytotoxic and other molecular targeted therapies whilst inducing a reversible cell cycle arrest in normal cells (reviewed by (Van Maerken et al., 2009a); (Vassilev, 2004; Shangary et al., 2008; Korotchkina et al., 2009; Cheok et al., 2011)). In recent years, there has been increased interest in the use of MDM2-p53 antagonists in a cyclotherapeutic setting to protect normal cells from the harmful effects of chemotherapy (reviewed by Cheok et al., 2011; van Leeuwen, 2012) and also in the identification of natural MDM2 inhibitors (Qin et al., 2012). It is worth mentioning here that despite structural similarities between MDM2 and MDMX, MDM2-p53 antagonists are largely ineffective against MDMX, and in some cases overexpression of MDMX has been reported to confer resistance to MDM2 antagonists (reviewed by Wade and Wahl, 2009).

Evaluation of Nutlin-3 and other MDM2-p53 antagonists in preclinical models of neuroblastoma have reported potent anti-tumor effects such as induction of growth arrest, senescence, differentiation and apoptosis, and inhibition of tumor cell proliferation and metastasis (Barbieri et al., 2006; Van Maerken et al., 2006, 2009a, 2011; Hardcastle et al., 2011; Patterson et al., 2011; Gamble et al., 2012). In addition, Nutlin-3 has been shown to sensitize neuroblastoma cells to chemotherapy induced apoptosis (Barbieri et al., 2006; Michaelis et al., 2009; Peirce and Findley, 2009a; Patterson et al., 2011). Furthermore, in line with observations that p53 is a direct target gene of MYCN and mediates MYCN induced apoptosis, we recently reported that MYCN sensitizes neuroblastoma cells to both Nutlin-3 and MI63 (Gamble et al., 2012). Moreover, there is also evidence that the *MDM2* and *p14^ARF^* status of p53 wt neuroblastoma cells can affect the response to MDM2-p53 antagonists and warrants further investigation (Van Maerken et al., 2011; Gamble et al., 2012).

Some studies to date have reported several p53-independent functions of Nutlin-3, including disruption of the MDM2-p73 interaction, which leads to enhanced p73 activity, suppression of tumor cell growth, and induction of apoptosis in p53 deficient neuroblastoma cells (Lau et al., 2008). Additionally, Nutlin-3 has also been shown to sensitize p53 deficient chemoresistant neuroblastoma cells to chemotherapy induced apoptosis via upregulation of TAp73 and E2F1 (Ambrosini et al., 2007; Peirce and Findley, 2009b), and inhibition of P-glycoprotein (Michaelis et al., 2009).

The low incidence of *p53* mutations in pediatric cancers, in particular neuroblastoma, compared with adult malignancies support the use of MDM2-p53 antagonists as a novel therapeutic strategy in the treatment of neuroblastoma. This is further strengthened by reports that the *MDM2* SNP309_T to G_ is associated with poor outcome, and that several mechanisms by which *MYCN* amplified neuroblastoma can overcome p53-mediated apoptosis are dependent on MDM2. Moreover, observations that inactivation of the p53/MDM2/p14^ARF^ pathway in relapsed neuroblastoma is predominantly due to lesions upstream of p53 combined with the reported therapeutic efficacy of Nutlin-3 in p53 wt multi-drug-resistant preclinical models of neuroblastoma with metastatic burden (Van Maerken et al., 2009a), highly support reactivation of p53 by inhibiting MDM2 as an attractive treatment option for metastatic relapsed neuroblastoma. MDM2-p53 antagonists are currently undergoing phase I clinical evaluation in adults and are anticipated to enter pediatric clinical trials in the near future, including neuroblastoma. There is however recent evidence in neuroblastoma cell lines suggesting that continuous exposure to Nutlin-3 can induce *de novo* *p53* mutations, resulting in cells exhibiting Nutlin-3 and multi-drug resistance. Consequently these results suggest that patients treated with MDM2-p53 antagonists should be closely monitored for the development of *p53* mutations and/or that MDM2-p53 antagonists should be given in combination with other agents to try to prevent the development of p53 mutations (Michaelis et al., 2011).

#### Aurora kinase inhibitors

The Aurora kinase family which includes Aurora A, B, and C, have attracted much attention over recent years as potential novel anti-cancer targets. In particular, AURKA has been shown to have oncogenic properties and is amplified and/or overexpressed in a range of human cancers (Maris, 2009). In neuroblastoma AURKA has been found to be expressed at high levels in *MYCN* amplified tumors and required for the growth of *MYCN* amplified cells (Berwanger et al., 2002; Otto et al., 2009). Studies have shown that it suppresses p53 transcriptional activity as well as promoting increased MDM2-mediated p53 degradation (Katayama et al., 2004; Liu et al., 2004). Additionally, via direct interaction but in a kinase independent manner, AURKA has been reported to stabilize MYCN by preventing its ubiquitin mediated degradation (Maris, 2009; Otto et al., 2009). These observations support the therapeutic potential of using AURKA inhibitors to simultaneously restore p53 activity and destabilize MYCN in neuroblastoma. Consistent with this, inhibition of AURKA expression in neuroblastoma cells has been shown to lead to growth inhibition, increased p53 expression, and decreased MYCN expression (Otto et al., 2009). Preclinical evaluations of a second generation AURKA inhibitor MLN8237 in pediatric cancers including neuroblastoma have been promising (Maris et al., 2010; Carol et al., 2011). Additionally, Aurora kinase inhibitor CCT137690 has also shown preclinical efficacy, downregulating MYCN, upregulating p53, and inhibiting neuroblastoma tumor growth *in vitro* and *in vivo*. Furthermore, consistent with the observation that AURKA is required for growth of *MYCN* amplified neuroblastoma, cells expressing high levels of MYCN were more sensitive to CCT137690 in comparison to cells expressing low or no MYCN (Faisal et al., 2011). Several AURKA inhibitors are currently undergoing clinical evaluations for use as a single agent or in combination with existing chemotherapeutics in various phase I-II trials for different human cancers including neuroblastoma (Cheung et al., 2011). Interestingly, the concomitant inhibition of the MDM2-p53 interaction and Aurora kinases has been shown to act synergistically to induce apoptosis in leukemia cells (Kojima et al., 2008), and should be assessed in neuroblastoma.

#### Twist-1 and BMI-1 inhibitors

Twist-1 and BMI-1 are involved in inactivation of the p53 pathway and are overexpressed in several cancers including neuroblastoma, often correlating with aggressive disease, and poor prognosis (reviewed by Wu et al., 2012a). Strategies which can inhibit Twist-1 and/or BMI-1 activity or expression and lead to restoration of the p53 pathway and tumor suppression are therefore therapeutically promising. In support of this, studies in neuroblastoma have observed that MYCN functionally cooperates with Twist-1 or BMI-1 to induce neuroblastoma tumorigenesis, where overexpression of Twist-1 or BMI-1 is necessary for tumor growth both *in vitro* and *in vivo* (Valsesia-Wittmann et al., 2004; Cui et al., 2007; Huang et al., 2011b). To date no specific inhibitor of Twist-1 has been identified, however PTC Therapeutics Inc., has very recently announced the development of PTC596, a first in its class, potent, oral, and selective inhibitor of BMI-1 protein expression. The inhibitor acts by altering the post-translational modification of BMI-1 which results in reduced levels of the protein and subsequently the reduced action of epigenetic complexes dependent on BMI-1^4^. In addition, histone deacetylase (HDAC) inhibitors and the drug artemisinin have also been shown to reduce expression levels of BMI-1 (Cao et al., 2011). Furthermore, as Twist-1 and BMI-1 both act through negative regulation of p14^ARF^ to inactivate the p53 pathway, this could also be overcome by the use of MDM2-p53 antagonists. A study to evaluate the therapeutic efficacy of PTC596 in neuroblastoma is warranted.

#### miRNA mimics and antagomirs

It has become increasingly evident that miRNAs play a role in tumorigenesis and thus are potential targets for cancer therapy. The widespread deregulation of miRNAs in neuroblastoma is reported to be due to *MYCN* amplification and chromosomal imbalances (Bray et al., 2009). Several miRNAs have been suggested to have prognostic significance and therapeutic potential in neuroblastoma including *miR-34a* and *miR-380-5p* (reviewed by Verissimo et al., 2011). The restoration of *miR-34a* and inhibition of *miR-380-5p* have been shown to reactivate the p53 pathway and inhibit MYCN expression, as well as inhibiting tumor growth in cell lines and orthotopic murine models of neuroblastoma (Wei et al., 2008; Swarbrick et al., 2010; Tivnan et al., 2012). Advances in miRNA cancer therapy toward clinical use are being made. *MiR-34* mimics developed by Mirna Therapuetics Inc., are anticipated to enter clinical trials in early 2013 (Bader, 2012). Miravirsen, a miRNA-targeted drug inhibiting *miR-122*, was the first of its class to enter clinical trials and is currently in phase II trials for the treatment of patients with Hepatitis C virus (Hu et al., 2012).

#### Other strategies

Similar to *miR-34a*, *CHD5* is also located at 1p36, and is frequently deleted and/or methylated in several human cancers including neuroblastoma. 1p deletion and epigenetic silencing of *CHD5* have been suggested to account for the low expression observed in both neuroblastoma tumors and cell lines, as homozygous deletions or mutations were reported to be infrequent (Fujita et al., 2008; Koyama et al., 2012). The promising therapeutic implications of restoring *CHD5* expression in neuroblastoma has previously been shown by the reduced clonogenicity and xenograft tumor growth of neuroblastoma cell lines stably transfected with *CHD5* cDNA (Fujita et al., 2008). Very recently, genistein has been reported to demethylate the *CHD5* promoter, enhance the expression of CHD5 and p53, and inhibit neuroblastoma growth *in vivo* (Li et al., 2012b).

Originally identified as genes downregulated by MYCN, NDRG1, and NDRG2 have subsequently been shown to be necessary for p53-mediated apoptosis. NDRG1 has been shown to be upregulated in response to differentiation and suppress metastasis, and there is growing interest in using NDRG1 as a biomarker of disease progression (reviewed by Ellen et al., 2008). Very recently, NDRG1 has been shown to upregulate NEDD4L, PTEN, and SMAD4 and inhibit the PI3K and Ras signaling pathways, thereby implicating its involvement in regulating key oncogenic pathways (Kovacevic et al., 2012). The exact role of NDRG1 and family members in cancers including neuroblastoma remain to be fully elucidated, however the iron chelator Dp44mT which exhibits anti-cancer properties has been reported to upregulate the expression of NDRG1 (Chen et al., 2012).

## SKP2

S-phase kinase-associated protein 2 (SKP2) is an F-Box protein and the variable substrate recognition component of the E3 ubiquitin ligase SCF^SKP2^ complex. The invariable core components of SCF complexes are SKP1, CUL1, and RBX1 (Skowyra et al., 1997; Deshaies, 1999; Nakayama and Nakayama, 2005). SKP2 was originally discovered as a protein associated with Cyclin A-CDK2, and subsequently shown to play a key role in promoting cell cycle progression, in particular at the G_1_/S transition (Zhang et al., 1995). More recently, studies have also identified the involvement of SKP2 in several processes closely linked to tumorigenesis such as cell survival, apoptosis, migration, and metastasis (reviewed by Chan et al., 2010b).

SKP2 protein is approximately 45 kDa, consisting of an N-terminal F-Box domain which mediates the interaction between SKP2 and SKP1, thereby tethering SKP2 to the SCF complex, and C-terminal leucine-rich repeats (LRR) which enable SKP2 to directly bind to target substrates (Bai et al., 1996; Skowyra et al., 1997; Schulman et al., 2000; Zheng et al., 2002). Since its discovery, SKP2 has been found to target numerous proteins for ubiquitination and subsequent degradation via the 26S proteasome pathway, including CDK inhibitors p21^CIP1^ (Yu et al., 1998; Bornstein et al., 2003), p27^KIP1^ (Carrano et al., 1999; Sutterluty et al., 1999; Tsvetkov et al., 1999), and p57^KIP2^ (Kamura et al., 2003), Rb family member p130 (Tedesco et al., 2002), apoptosis regulator FOXO1, tumor suppressors BRCA2 (Moro et al., 2006), RASSF1A (Song et al., 2008), and TOB1 (Hiramatsu et al., 2006), Cyclins D (Yu et al., 1998) and E (Yeh et al., 2001), as well as oncogenes c-MYC (Kim et al., 2003; von der Lehr et al., 2003) and MYB (Charrasse et al., 2000). The binding and recognition of the substrate by SKP2 is dependent on prior phosphorylation of the target substrate, and in some cases also requires the activity of a co-factor protein, CKS1 (Cyclin-kinase-subunit 1; Ganoth et al., 2001; Spruck et al., 2001; Frescas and Pagano, 2008).

### Regulation of SKP2 expression, stability, and activity

Consistent with its role as a cell cycle regulator, expression levels of SKP2 oscillate during the cell cycle, and are regulated by both transcriptional and post-transcriptional mechanisms (Bashir and Pagano, 2004). Levels of SKP2 are low in G_0_/G_1_ and late M/early G_1_, increase during G_1_/S transition and reach a maximum in S phase (Wirbelauer et al., 2000; Bashir et al., 2004; Wei et al., 2004). Studies to date have revealed that several transcription factors act directly via the *SKP2* promoter to upregulate *SKP2* gene expression, such as E2F1 (Zhang and Wang, 2006), NFkB (Schneider et al., 2006), SP1 (Appleman et al., 2006), CBF1 (Sarmento et al., 2005), GABP (Imaki et al., 2003), FOXM1 (Wang et al., 2005), c-MYC (Bretones et al., 2011), STAT3 (Huang et al., 2012), and NOR1 (Gizard et al., 2011). In addition, Foxp3 (Zuo et al., 2007), FOXO3A (Wu et al., 2012b) and STAT1 (Wang et al., 2010c) have been identified to transcriptionally repress *SKP2* expression.

The identification of *SKP2* as a direct target gene of E2F (Zhang and Wang, 2006) is of particular importance as Yung et al. (2007) subsequently described the SKP2 autoinduction loop comprising pRB-E2F, SKP2, p27^KIP1^, and Cyclin E-CDK2, in which SKP2 expression initiates proteolysis of p27^KIP1^, activation of Cyclin E-CDK2 which feeds back to sustain pRB inactivation, E2F release, and further induction of the *SKP2* gene. Therefore, once activated, this autoinduction loop ensures mitogen independent cell cycle progression through the restriction point.

In addition to transcriptional regulation, SKP2 is also subject to regulation via protein stability. The E3 ubiquitin ligase APC^Cdh1^ complex mediates the ubiquitination and subsequent degradation of SKP2, primarily in early G_1_ (Bashir et al., 2004; Wei et al., 2004). The interaction between Cdh1 and SKP2 is essential for APC^Cdh1^ complex mediated degradation of SKP2. In support of this, several kinases including CDK2, AKT, and Pim-1 have been shown to phosphorylate SKP2 at residues Ser-64 and Ser-72 and/or Thr-417, thereby inhibiting the Cdh1-SKP2 interaction and protecting SKP2 from APC^Cdh1^ mediated ubiquitination and degradation. Furthermore, Pim-1 has also been shown to phosphorylate Cdh1, impairing its association with CDC27 and inhibiting APC^Cdh1^ activity, thereby protecting SKP2 from degradation (reviewed by Chan et al., 2010b). In contrast, studies have shown that pRb interacts with Cdh1 and promotes SKP2 degradation (Binne et al., 2007), and TGFβ signaling induces nuclear translocation of SKP2 which facilitates its ubiquitylation by APC^Cdh1^ and subsequent degradation (Hu et al., 2011).

Finally, the formation of the SCF^SKP2^ complex is critical for ligase activity. Studies have demonstrated that PI3K/AKT signaling pathway and Cyclin D positively regulate SCF^SKP2^ complex formation and ligase activity, potentially through neddylation of CUL1 (reviewed by Chan et al., 2010b).

### SKP2 as an oncoprotein

Numerous studies to date have provided evidence showing the oncogenic potential of SKP2, and its cross-talk with multiple signaling pathways involved in carcinogenesis such as PI3K/AKT, mTOR, ERK, NFkB, Ras/MAPK, and IGF (Wang et al., 2012c). Overexpression of SKP2 can drive quiescent cells to enter the cell cycle (Sutterluty et al., 1999), and promote adhesion-independent growth of cancer cells (Carrano and Pagano, 2001; Signoretti et al., 2002). Downregulation or inhibition of SKP2 expression leads to growth arrest and/or apoptosis, as well as reduced cell migration, invasion, and metastasis (Koga et al., 2003; Yokoi et al., 2003; Jiang et al., 2005; Lee and McCormick, 2005; Shibahara et al., 2005; Katagiri et al., 2006; Kitagawa et al., 2008; Xiao et al., 2009; Chan et al., 2010a; Bretones et al., 2011). In transgenic mouse models, SKP2 cooperates with N-Ras to drive lymphomagenesis (Latres et al., 2001), and tissue targeted expression of *SKP2* results in hyperplasia, dysplasia, and low-grade carcinoma of the prostate gland (Shim et al., 2003). Knockdown of *SKP2* significantly reduced tumor formation and metastasis of breast cancer xenografts (Chan et al., 2010a). Furthermore, work in *SKP2* knockout mice demonstrated that SKP2 is necessary for tumor formation induced by *PTEN*, *p19^ARF^*, or *Rb* deficiency (Lin et al., 2010; Wang et al., 2010b).

SKP2 overexpression at the mRNA and/or protein level have been detected in a number of human tumors and cell lines including prostate, breast, pancreatic, gastric, colorectal, ovarian, melanoma, lymphoma, and leukemia (reviewed by Nakayama and Nakayama, 2006; Frescas and Pagano, 2008; Hershko, 2008). In addition, amplification of *SKP2* at chromosome 5p13 has been reported (Yokoi et al., 2004; Saigusa et al., 2005; Wang et al., 2009; Rose et al., 2011; Li et al., 2012a), and tends to be observed in metastatic tumors whereas overexpression of SKP2 is reported in early cancers (Hershko, 2008). Overall, elevated SKP2 expression has been shown to correlate with a poor prognostic outcome, tumor size, dedifferentiation, advanced grade, and metastasis (reviewed by Wang et al., 2012c). It is worth mentioning that in some cases the latter has been correlated with cytoplasmic and not nuclear SKP2 expression. In the vast majority of cases, SKP2 overexpression inversely correlates with p27^KIP1^ expression (Nakayama and Nakayama, 2006; Frescas and Pagano, 2008), which is consistent p27^KIP1^ being a key target of SKP2 and to be rarely mutated in cancer (Chu et al., 2008). Finally, in recent years, SKP2 overexpression has been shown to mediate resistance to TRAIL induced apoptosis (Schuler et al., 2011), and radio- and chemoresistance of human cancer cells (Ishii et al., 2004; Davidovich et al., 2008; Chan et al., 2012; Totary-Jain et al., 2012; Wang et al., 2012b).

#### SKP2 mediated attenuation of p53 function

**Figure 3:**
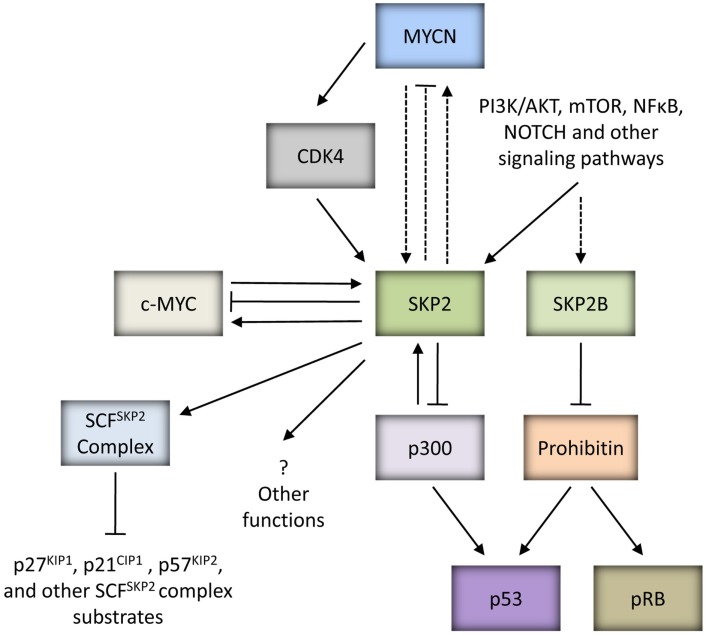
**MYC proteins and SKP2**. SKP2 is an oncoprotein which can be upregulated by MYC proteins to drive tumorigenesis. *SKP2* is a direct target gene of c-MYC, and can regulate the stability of c-MYC and be a co-factor for c-MYC mediated transcriptional activation of target genes. Due to the homology between c-MYC and MYCN, it is possible that *SKP2* is also a direct target gene of MYCN and plays a similar role in regulating MYCN stability and transactivation of MYCN target genes (as indicated by the dashed lines). In neuroblastoma, MYCN can indirectly upregulate *SKP2* via CDK4. In addition to upregulation by oncogenic MYC proteins, several signaling pathways closely linked to carcinogenesis such as PI3K/AKT and mTOR have been shown to influence SKP2 expression, stability and SCF^SKP2^ ligase activity. SKP2 is a component of the SCF^SKP2^ complex which mediates the degradation of several substrates including CDK inhibitors p21^CIP1^, p27^KIP1^, and p57^KIP2^. Independently of SCF complex formation, SKP2 can bind to p300 and attenuate p53 function. Interestingly, p300 is able to reciprocally regulate SKP2 activity. SKP2B, an alternatively spliced variant of *SKP2*, can perturb both p53 and pRB pathways via degradation of Prohibitin. It is possible that SKP2 has other functions which may promote tumorigenesis.

Recent key publications have identified that SKP2 and an alternatively spliced variant of *SKP2*, SKP2B can attenuate p53 function through independent mechanisms (Figure 3). Initially, Kitagawa et al. (2008) identified that SKP2 is able to suppress p53-mediated apoptosis by inhibiting the p53 transcriptional co-activator, p300. This function was shown to be independent of the F-Box motif of SKP2 and hence SCF^SKP2^ complex function. SKP2 was found to bind to the CH1 and CH3 domain of p300, thought to be p53 binding sites, thereby antagonizing the p53-p300 interaction, suppressing p300-mediated acetylation of p53 and the transactivation ability of p53. In addition, secondary to the latter, SKP2 was also observed to promote the degradation of p300. As proof-of-concept, the study demonstrated that inhibition of SKP2 combined with DNA damaging agents or Nutlin-3, led to greater induction of apoptosis (Kitagawa et al., 2008). More recently, SKP2B was demonstrated to attenuate p53 function via a mechanism independent of p300. SKP2B is shown to bind to and mediate the degradation of Prohibitin, a potential TSG which binds to and stimulates p53 transcriptional activity. Taken together, these observations suggest that amplification of the *SKP2* locus would represent a powerful mechanism to attenuate p53 function in cancer (Chander et al., 2010; Figure 3).

Interestingly, a very recent study has reported an additional mechanism for the SKP2-p300 interaction, whereby p300 is able to reciprocally regulate SKP2 function (Figure 3). p300-mediated acetylation of SKP2 at Lys-68 and Lys-71 within its NLS region was shown to promote SKP2 stabilization and cytoplasmic retention, thereby influencing its oncogenic potential. In part the latter was reported to be achieved through cytoplasmic SKP2 mediated increased cellular migration via ubiquitination and destruction of E-cadherin (Inuzuka et al., 2012).

### The role of SKP2 in neuroblastoma

In line with observations that SKP2 drives cellular proliferation, cAMP induces proliferation of neuroblastoma cells by upregulating SKP2 (Cho et al., 2007), while growth arrest and differentiation of neuroblastoma cell lines induced by retinoic acid, BMP2, and the HDAC inhibitor *Helminthosporium carbonum* toxin is accompanied by a decrease in SKP2 levels (Nakamura et al., 2003a,b; Cuende et al., 2008; Deubzer et al., 2008a). This decrease has been attributed to retinoic acid induced downregulation of Rae1 which leads to increased APC^Cdh1^ mediated degradation of SKP2, or HDAC inhibitor induced activation of the Rb pathway (Cuende et al., 2008; Deubzer et al., 2008b).

Several studies including our own have found higher expression of SKP2 in the presence of ectopic MYCN, and in *MYCN* amplified cell lines and tumors (Sugihara et al., 2006; Bell et al., 2007a; Westermann et al., 2007; Chen et al., 2010; Muth et al., 2010; Table 2), suggesting that deregulated SKP2 expression is driven by MYCN to promote neuroblastoma. The exact role of SKP2 in neuroblastoma pathogenesis is currently unclear. It is possible that high expression of SKP2 simply collaborates with MYCN to drive uncontrolled proliferation through increased degradation of CDK inhibitors. Alternatively, as *Myc* family members are known to drive both proliferation and apoptosis, MYCN may upregulate SKP2 to inhibit apoptosis and tip the balance in favor of proliferation. In particular, as *p53* has previously been shown to be a direct target gene of MYCN, and a mechanism for MYCN mediated apoptosis in neuroblastoma, it is plausible that MYCN directly upregulates SKP2 to attenuate p53-mediated apoptosis (Kitagawa et al., 2008; Chen et al., 2010), as has previously been reported for c-MYC (Bretones et al., 2011; Figure 3).

**Table 2:** **Summary of studies which have shown increased SKP2 expression in the presence of MYCN and/or *MYCN* amplification in neuroblastoma**.

Study	Tumors	Cell Lines	Methods	Evidence
Sugihara et al. (2006)	–	SHEP-MYCN^1^	WB	E
Bell et al. (2007a)	–	IMR32, SKNBE2C, Tet21N^2^	MYCN siRNA, microarray^3^, qRT-PCR	Si, E
Westermann et al. (2007)	49/117/141/19
	Microarray/qRT-PCR/IHC tissue array/WB	–	Microarray, qRT-PCR, WB, IHC	T
Chen et al. (2010)	–	Tet21N	Microarray	E
Muth et al. (2010)	251 Microarray	SHEP, SHSY5Y, SKNSH, SKNBE2C, IMR32, IMR5-75, Kelly, Tet21N, IMR5-75-shMYCN^4^	Microarray, SKP2 promoter assay, MYCN shRNA, qRT-PCR, WB	T, R, Sh, E

*^1^SHEP cells transfected with retroviral control vector or vector containing MYCN; ^2^GIMEN, NB69, LAN-6, NBL-S, PER-108, SMSKCNR, NBLW, CHLA-136, and NGP were analyzed for SKP2 protein expression using WB, however no correlation between SKP2 protein expression and MYCN protein expression or *MYCN* amplification was observed; ^3^Microarray analysis performed on IMR32 and SKNBE2C cells treated with scrambled or MYCN siRNA for 16 and 48 h; ^4^IMR5-75 cells with inducible stably transfected short hairpin RNAs against MYCN*.

*WB, Western blotting; IHC, immunohistochemistry; R, reporter gene assays; Si, differential expression following MYCN siRNA; Sh, differential expression following MYCN shRNA; E, differential expression/correlation in ectopic MYCN expression systems; T, correlation in tumors with *MYCN* amplification*.

The precise mechanism of MYCN mediated upregulation of SKP2 remains unclear, as *SKP2* is a direct target gene of E2F (Zhang and Wang, 2006), *E2F* is a direct target of c-MYC (Fernandez et al., 2003) and higher E2F expression levels are observed in the presence of MYCN (Mac et al., 2000; Woo et al., 2008). In neuroblastoma tumors, *SKP2* expression has been shown to tightly correlate with *E2F1* expression, and both genes are expressed at significantly higher levels in *MYCN* amplified tumors (Westermann et al., 2007; Muth et al., 2010). Interestingly, although *SKP2* expression levels were found to be highest in *MYCN* amplified tumors, this did not correlate with *MYCN* expression in *MYCN* single-copy tumors (Westermann et al., 2007). Similarly, Bell et al. (2007a) reported that although *SKP2* mRNA levels decreased after MYCN siRNA and after removal of MYCN expression in Tet21N cells, SKP2 protein levels did not correlate with MYCN protein levels or *MYCN* amplification in a panel of neuroblastoma cell lines. It has been suggested that deregulated MYCN expression in *MYCN* amplified neuroblastoma indirectly upregulates *SKP2* via the induction of *CDK4* (Figure 3), thereby reducing the abundance of repressive pRB-E2F1 complexes bound to the *SKP2* promoter and providing an entry point into the SKP2 autoinduction loop (Muth et al., 2010). Whether *SKP2* is a direct transcriptional target of MYCN remains to be determined. However, as c-MYC and MYCN are known to share some common target genes, and c-MYC has recently been shown to directly bind to two high affinity E-Box motifs (CATGCG and CACGCG) mapping to −756 and −389 bp upstream of the transcriptional start site within the *SKP2* promoter and drive transcription (Bretones et al., 2011), it is therefore possible that MYCN may also directly regulate *SKP2* expression (Figure 3).

Whether or not *SKP2* is a direct target gene of MYCN, the prognostic significance of *SKP2* overexpression in neuroblastoma has been demonstrated in primary tumor samples. High *SKP2* expression was found to characterize aggressive high-risk disease independent of both *MYCN* status and stage. Furthermore, high SKP2 expression was observed to correlate with low p27^KIP1^ expression levels (Westermann et al., 2007), consistent with studies in other cancer types (reviewed by Nakayama and Nakayama, 2006; Frescas and Pagano, 2008; Hershko, 2008). Interestingly, the prognostic value of low p27^KIP1^ expression in neuroblastoma, independent of *MYCN* status had previously been demonstrated (Bergmann et al., 2001).

SKP2 has previously been shown to regulate the stability of c-MYC and to be a co-factor for c-MYC mediated transcriptional activation of target genes (Kim et al., 2003; von der Lehr et al., 2003), however it is currently remains unclear whether it plays a similar role in regulating MYCN stability and transactivation of MYCN target genes (Figure 3).

Taken together, SKP2 has prognostic significance in neuroblastoma and could be a promising novel therapeutic target, however further studies investigating the role of SKP2 in neuroblastoma are required. Consequently, together with *in vitro* experiments, crossing *SKP2* knockout mice with murine models of neuroblastoma, such as the transgenic TH-*MYCN* mouse, the role of SKP2 in driving neuroblastoma could be elucidated.

### Therapeutic strategies targeting SKP2

Over the past decade, SKP2 has emerged as a novel and attractive pharmacological target in cancer therapeutics. This is supported by the involvement of SKP2 in proliferation, apoptosis, differentiation, migration, and metastasis, as well as its cross-talk with multiple signaling pathways closely linked to carcinogenesis. In addition, the prognostic significance and overexpression of SKP2 in a variety of human cancers, combined with observations that *SKP2* knockout mice are both viable and fertile (Nakayama et al., 2000), further strengthens its appeal. Furthermore, with the discovery of the role of SKP2 and SKP2B in attenuating p53-mediated apoptosis and transcriptional activity, and that *SKP2* deficiency triggers a potent ARF-p53-independent cellular senescence in the presence of oncogenic conditions (such as inactivation of TSGs/overexpression of proto-oncogenes), has implicated the wide applicability of targeting SKP2 as a strategy to reactivate p53 and as pro-senescence therapy (Kitagawa et al., 2008; Chander et al., 2010; Lin et al., 2010). Finally, the availability of crystallographic maps of the structure of SKP2 and SCF^SKP2^ complex components and their interaction with substrates, together with biochemical data provides the opportunity to develop novel SKP2 inhibitors (Frescas and Pagano, 2008).

Inhibitors targeting the ubiquitin proteasome system (UPS) have been developed and used with success in preclinical and/or clinical studies thereby highlighting these inhibitors as a new class of cancer therapeutics (Kisselev et al., 2012). Bortezomib (Velcade^™^) is the first clinically approved proteasome inhibitor, and has been reported to induce p27^KIP1^ expression through degradation of SKP2 (Uddin et al., 2008, 2009). Of interest, although not directly linked to SKP2, bortezomib is shown to induce apoptosis and inhibit cell growth, migration, angiogenesis, and metastasis both *in vitro* and in murine models of chemosensitive and chemoresistant neuroblastoma (Brignole et al., 2006; Michaelis et al., 2006; Hamner et al., 2007; Valentiner et al., 2009). Despite the overall success of bortezomib, severe side-effects have been reported in patients, and may be attributable to the broad ranging functions and targets of the UPS (Chen et al., 2011). Therefore the development of specific inhibitors directly targeting SKP2 is likely to have enhanced efficacy and reduced toxicity.

To date, no specific inhibitor of SKP2 has been identified however three SMIs have been shown to downregulate SKP2 activity, and exhibit anti-tumor effects in preclinical studies. Using high-throughput screening, Cpd A and SMIP004 were identified (Chen et al., 2008; Rico-Bautista et al., 2010). CpdA, interferes with the SKP1/SKP2 interaction, thereby directly preventing SCF^SKP2^ complex formation and ligase activity that results in the accumulation of several SCF^SKP2^ substrates including p27^KIP1^ and p21^CIP1^. It has been shown to induce cell cycle arrest and apoptosis, exhibiting preferential activity against neoplastic cells, as well as overcoming chemoresistance to dexamethasone, doxorubicin, melphalan, and bortezomib (Chen et al., 2008). Similarly, SMIP0004 was identified and shown to inhibit cell growth and induce apoptosis, and exhibit cancer cell specificity. It was found not only to downregulate SKP2 and induce dose-dependent stabilization of p27^KIP1^ and p21^CIP1^, but also to inhibit cellular CDK2 activity (Rico-Bautista et al., 2010). Finally, MLN4924 is a compound which targets NEDD8-activating enzyme, consequently affecting CUL1 neddylation and impairing SCF^SKP2^ complex formation and ligase activity. Preclinically, MLN4924 exhibits potent cytotoxicity against a panel of human cancer cell lines, and suppresses tumor growth in mouse xenograft models (Soucy et al., 2009; Lin et al., 2010). Furthermore, in contrast to bortezomib, MLN4924 did not significantly affect bulk protein turnover (Soucy et al., 2009). MLN4924 was recently evaluated by the Pediatric Preclinical Testing Program (PPTP), exhibiting potent activity *in vitro* and inhibiting tumor growth against a subset of the PPTP solid tumor and ALL xenografts. Specifically in the neuroblastoma panel, MLN4924 had a median relative IC_50_ of 278nM, and induced intermediate activity (EFS T/C values >2) in 1/4 xenografts (Smith et al., 2012). In addition to the above SMIs, several natural compounds have also been shown to downregulate SKP2 activity, including retinoic acid, silibinin, curcumin, quercetin, lycopene, epigallocatechin-3-gallate (Nakamura et al., 2003a; Roy et al., 2007; Cuende et al., 2008; Huang et al., 2011a). Retinoic acid is of particular relevance to neuroblastoma, as Isotretinoin (13-*cis* RA) is routinely used in the treatment of high-risk disease following high-dose chemotherapy and stem cell rescue, in a setting of minimal residual disease (Wagner and Danks, 2009).

Several signaling pathways have been shown to influence SKP2 expression, stability, and SCF^SKP2^ ligase activity, therefore an alternative strategy would be to use inhibitors that target components of these pathways such as PI3K/AKT, mTOR, ERK, IGF, Notch, and IKK/NFkB, and some of which are currently in clinical trials. In support of this, PI3K inhibitor LY294002 and Rapamycin have been shown to decrease SKP2 expression (Motti et al., 2005; Shapira et al., 2006). Of interest, it is worth mentioning that LY294002 has also been shown to destabilize MYCN in neuroblastoma (Chesler et al., 2006). Targeting the expression or stability of SKP2 is an appealing option due to the oncogenic functions of SKP2 which are independent of SCF^SKP2^ ligase formation and activity (Ji et al., 2006; Kitagawa et al., 2008; Chan et al., 2010a).

## DKK3

The Wnt signaling pathway is involved in embryogenesis, playing a key role in both early and late cell-fate decisions, and also in adult tissue homeostasis. Deregulated activation of this pathway has been implicated in several malignancies (Klaus and Birchmeier, 2008). *DKK3* is a member of the *Dickkopf* family of secreted Wnt antagonists, which also includes *DKK1*, −*2*, −*4* and *DKKL1*. *DKK* family members encode glycoproteins which all share an N-terminal signal peptide, and with the exception of DKKL1, also share two cysteine-rich domains termed Cys1 and Cys2 that are separated by a non-conserved linker region of variable length. DKK3 has a molecular weight of 38 kDa, and Cys1 and Cys2 are separated by a linker region of 13 amino acids (reviewed by Veeck and Dahl, 2012).

DKK1, −2 and −4, are well-established antagonists of the canonical Wnt/β-catenin pathway through direct high affinity binding to Wnt co-receptors lipoprotein receptor-related protein 5 and 6 (LRP5/6), and also DKK receptors Kremen 1 and 2 (KRM1/2; reviewed by (Niehrs, 2006)). The co-binding of DKK proteins to KRM1/2 receptors potentiates the ability of DKK proteins to inhibit Wnt signaling, through the formation of a ternary complex that leads to endocytosis and degradation of LRP receptors (Mao et al., 2002; Mao and Niehrs, 2003). In contrast, the role of DKK3 as a Wnt inhibitor is less clear. In some studies, DKK3 is not shown to bind to LRP5/6 at the cell surface membrane, and whether it binds to KRM1/2 remains controversial (Mao et al., 2002; Nakamura and Hackam, 2010). In contrast, others have however confirmed DKK3 as an inhibitor of the canonical pathway (Yue et al., 2008; Lee et al., 2009; Dellinger et al., 2012).

### DKK3 as a tumor suppressor

Several lines of evidence exist to suggest that DKK3 acts as a tumor suppressor. Originally identified as a novel TSG by Tsuji et al. (2000) using an *in vitro* transformation model of normal human fibroblasts, reduced DKK3 expression was subsequently observed in cell lines and tumors of several different cancer types including liver, lung, prostate, breast, osteosarcoma, and leukemia (Tsuji et al., 2000; Nozaki et al., 2001; Hsieh et al., 2004; Kurose et al., 2004; Roman-Gomez et al., 2004; Abarzua et al., 2005; Tanimoto et al., 2007; Mizobuchi et al., 2008; Veeck et al., 2008; Yue et al., 2008; Yu et al., 2009; Dellinger et al., 2012). Studies into the mechanisms behind reduced DKK3 expression have revealed histone modification in cancers such as renal cell carcinoma (Ueno et al., 2011), and hypermethylation of the *DKK3* promoter in a vast number of human cancers including lung, bladder, breast, and leukemia (reviewed by Veeck and Dahl, 2012). Additionally, loss of heterozygousity and homozygous deletions of the *DKK3* gene at 11p15 have also been reported (Tsuji et al., 2001; Bashyam et al., 2005). In some cases, reduced DKK3 expression and/or *DKK3* promoter methylation has been shown to be associated with poor prognostic clinicopathologic characteristics and outcome (Roman-Gomez et al., 2004; Yue et al., 2008; Veeck et al., 2009; Yu et al., 2009; Yang et al., 2010; Dellinger et al., 2012; Wang et al., 2012a).

Overexpression of DKK3 has been shown to mediate potent anti-tumor effects including reduced cell proliferation, anchorage-independent growth, and invasion and metastasis, and induced cancer cell specific apoptosis both *in vitro* and in murine tumor models (Tsuji et al., 2001; Abarzua et al., 2005; Edamura et al., 2007; Tanimoto et al., 2007; Koppen et al., 2008; Mizobuchi et al., 2008; Kawasaki et al., 2009; Gu et al., 2011; Than et al., 2011; Ueno et al., 2011; Dellinger et al., 2012; Yang et al., 2012). The precise mechanisms by which DKK3 inhibits tumorigenesis are not completely understood. Studies using a replication incompetent adenoviral vector incorporating the human *DKK3* cDNA (Ad-REIC/DKK3) have demonstrated that DKK3 induced apoptosis is mediated via ER stress triggered activation of JNK with subsequent reduction in anti-apoptotic BCL2, mitochondrial translocation of BAX, cytochrome C release, and activated caspase cleavage (Abarzua et al., 2005, 2007; Tanimoto et al., 2007; Kashiwakura et al., 2008; Kobayashi et al., 2008; Kawasaki et al., 2009). Ad-REIC/DKK3 has also been shown to induce an indirect host-mediated anti-tumor effect via the induction of IL-7 (Sakaguchi et al., 2009). It is however important to mention that mechanisms which mediate resistance to DKK3 induced apoptosis have been identified, such as the overexpression of heat shock protein 70/72, an ER-residing chaperone BiP/GRP78 BiP/GRP78 and BCL2 (Veeck and Dahl, 2012). Interestingly, despite the vast body of evidence suggesting a fundamental role for DKK3 in cancer development, *DKK3* knockout mice were both viable and fertile and were not observed to be more susceptible to cancer development (Barrantes Idel et al., 2006).

### The role of DKK3 in neuroblastoma

DKK3 is secreted in neuroblastoma cell lines, and in primary tumors is found to be expressed in the endothelium (Koppen et al., 2008; Haug et al., 2011). An initial study of DKK3 and neuroblastic tumors observed that *DKK3* expression was a marker of tumor differentiation, where the highest expression was observed in the most differentiated ganglioneuromas, and the lowest expression in the least differentiated neuroblastoma. Specifically in neuroblastoma tumors, low *DKK3* expression correlated significantly with poor prognosis, however this was not independent of *MYCN* amplification (Koppen et al., 2008). In a more recent study of 101 neuroblastic tumors including 88 neuroblastoma tumors, both high *DKK3* and *DKK2* expression correlated with good prognosis (Revet et al., 2010).

**Figure 4:**
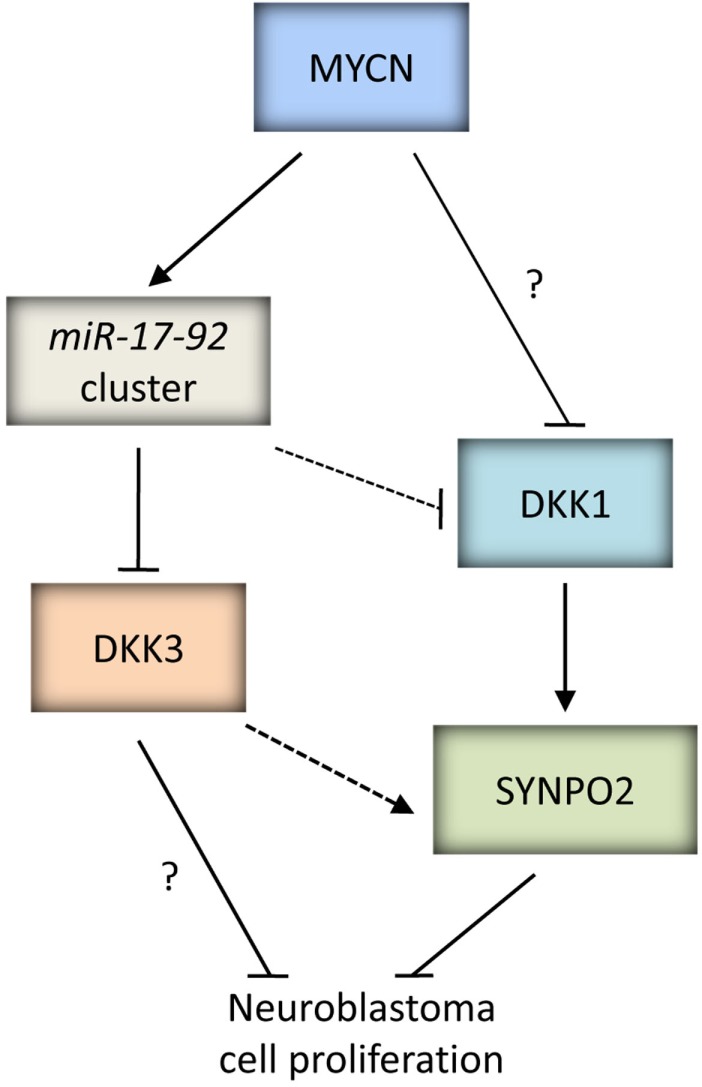
**MYCN mediated downregulation of DKK proteins in neuroblastoma**. DKK1 and DKK3 can inhibit the proliferation of neuroblastoma cells, therefore downregulation of DKK proteins by MYCN will favor neuroblastoma tumorigenesis. MYCN mediated downregulation of DKK3 can occur via upregulation of the oncogenic *miR-17-92* cluster, and due to the homology between *DKK* family members, this may also be a potential mechanism for MYCN mediated downregulation of DKK1 (as indicated by the dashed line). DKK1 inhibits the proliferation of neuroblastoma cells via upregulation of SYNPO2, and although the mechanism for DKK3 remains unclear, due to the homology between *DKK* family members this may also occur via SYNPO2 (as indicated by the dashed line).

Several studies to date including our own have observed an inverse relationship between DKK3 expression and *MYCN* amplification and/or expression in both cell lines and primary tumors, thereby suggesting that downregulation of DKK3 is necessary for progression of *MYCN* amplified neuroblastoma (Table 3; Bell et al., 2007a; Koppen et al., 2008; Chen et al., 2010; Haug et al., 2011; De Brouwer et al., 2012; Figure 4). Consistent with this, overexpression of DKK3 inhibited the proliferation of *MYCN* amplified neuroblastoma cell lines *in vitro* (Koppen et al., 2008).

**Table 3:** **Summary of studies which have observed an inverse relationship between DKK3 expression and MYCN expression and/or *MYCN* amplification in neuroblastoma**.

Study	Tumors	Cell Lines	Methods	Evidence
Bell et al. (2007a)	–	IMR32, SKNBE2C and Tet21N	MYCN siRNA, Microarray^1^, qRT-PCR	Si, E
Koppen et al. (2008)	13 Ganglioneuroma, 14 ganglioneuroblastoma, 82 neuroblastoma	14 MNA and 8 MYCN single-copy NB cell lines^2^, Tet21N, SKNAS-NMYCER	Microarray^3^, Northern blot	T, C, E
Chen et al. (2010)	–	Tet21N	Microarray	E
Haug et al. (2011)	25 Neuroblastoma	SKNBE2C, Kelly, Tet21N	IHC, shMYCN, qRT-PCR	T, Sh, E
De Brouwer et al. (2012)	101 Neuroblastoma	7 MNA and 5 MYCN single-copy NB cell lines^2^, Tet21N	qRT-PCR	T, C, E

*^1^Microarray analysis performed on IMR32 and SKNBE2C cells treated with scrambled or MYCN siRNA for 16 and 48 h; ^2^see publication for full list of cell lines used; ^3^performed on 12/22 neuroblastoma cell lines*.

*MNA; *MYCN* amplified; NB, neuroblastoma; IHC, immunohistochemistry; Si, differential expression following MYCN siRNA; Sh, differential expression following MYCN shRNA; E, differential expression/correlation in ectopic MYCN expression systems; T, differential expression/correlation in tumors with *MYCN* amplification; C, differential expression/correlation in cell lines with/without *MYCN* amplification*.

To determine the mechanism responsible for low DKK3 expression in *MYCN* amplified tumors, studies have observed that irrespective of *MYCN* status, reduced DKK3 expression in neuroblastoma was not due to promoter hypermethylation (Haug et al., 2011). Through ChIP analysis, MYCN was reported to downregulate *DKK3* expression through a mechanism independent of direct transcriptional repression (Koppen et al., 2008). Indeed, in support of this, two recent independent studies have shown that MYCN regulates the expression and secretion of DKK3 in neuroblastoma cells through direct upregulation of the oncogenic *miR-17-92* cluster (e.g., *miR-92a*, -*92b*, and -*19b*) which target the 3′UTR of *DKK3* (Haug et al., 2011; De Brouwer et al., 2012; Figure 4). Analysis of primary tumors demonstrated a positive correlation between *MYCN* expression and *miR-17-92* cluster expression, consistent with previous studies (Schulte et al., 2008), and an inverse correlation between *miR-17-92* cluster expression and *DKK3* expression (Haug et al., 2011; De Brouwer et al., 2012). In line with higher *DKK3* expression in ganglioneuroma and ganglioneuroblastoma, these tumors had lower levels of *miR-92a* compared with neuroblastoma (Haug et al., 2011).

The precise mechanism by which DKK3 suppresses neuroblastoma tumorigenesis is unclear however this does not appear to be through inhibition of the canonical Wnt/β-catenin signaling pathway (Koppen et al., 2008). It is possible that DKK3 may act by inhibiting non-canonical Wnt signaling pathways such as the Planar cell polarity (PCP)-pathway, or the Wnt/Ca^2+^ pathway. Interestingly, these non-canonical Wnt pathways have previously been shown to control neural crest migration (De Calisto et al., 2005).

Similar to DKK3, DKK1 is also reported to be secreted in neuroblastoma cell lines, and downregulated indirectly by MYCN (Koppen et al., 2007). Lower expression was observed in the presence of ectopic MYCN and in *MYCN* amplified tumors (Bell et al., 2007b; Koppen et al., 2007; Chen et al., 2010). Overexpression of DKK1 inhibited the proliferation of neuroblastoma cell lines through upregulation of synaptopodin-2 (SYNPO2), a protein previously reported to suppress tumor growth, and not by inhibition of canonical Wnt/β-catenin signaling (Koppen et al., 2007; Figure 4).

The above findings suggest that DKK proteins may play a role in neuroblastoma pathogenesis, therefore it would be interesting to assess tumor formation in TH-*MYCN* transgenic mice deficient in *DKK3* or *DKK1*. Certainly, the Wnt pathway is involved in early formation of the neural crest and the subsequent development, migration, and terminal differentiation of neural crest cells (Barembaum and Bronner-Fraser, 2005; Ille and Sommer, 2005), and Wnt/β-catenin signaling has been shown to directly induce *MYCN* expression (Kuwahara et al., 2010). Additionally, embryonal malignancies of childhood such as neuroblastoma have been suggested to be disorders of normal development (reviewed by Grimmer and Weiss, 2006; Johnsen et al., 2009), therefore it is not surprising that deregulated Wnt pathway signaling may be involved in neuroblastoma development.

To date, studies into Wnt signaling in neuroblastoma have shown that siRNA mediated inhibition of the Wnt1/β-catenin pathway leads to apoptosis of SHSY5Y cells (Zhang et al., 2009), and deregulated canonical and non-canonical Wnt signaling in chemoresistant and high-risk disease (Blanc et al., 2005; Liu et al., 2008b; Flahaut et al., 2009). Additionally, the expression of FZD6 is reported to predict poor survival in neuroblastoma patients, and marks rare and highly tumorigenic neuroblastoma stem cells (Cantilena et al., 2011). Furthermore, microarray analysis of Tet21N MYCN+ and MYCN− cells identified that in addition to *DKK1* and *DKK3*, there was deregulated expression of several components of the Wnt pathway in the presence of MYCN. These included downregulation of additional Wnt signaling antagonists *SFRP1* and *APC* together with the upregulated expression of positive regulators *FRAT2*, *CSNK2A1*, and *RUVBL1* (Chen, 2009; Chen et al., 2010). Interestingly, in an independent study, *SFRP1* was observed to correlate with good prognosis in neuroblastic tumors (Revet et al., 2010). *CSNK2A1* and *RUVBL1* have been reported to play multiple roles in driving malignant transformation (Duncan and Litchfield, 2008; Huber et al., 2008), and *RUVBL1* expression was identified to be associated with poor overall survival in neuroblastoma patients (Westermann et al., 2008). Finally, several upstream components of the Wnt pathway have been reported to be highly expressed in neuroblastic tumors and cell lines (Revet et al., 2010). Taken together, these findings provide support to suggest that the Wnt pathway may be important in neuroblastoma pathogenesis although the precise role remains unclear and will require further investigation.

### Therapeutic strategies to re-establish DKK3

Developmental pathways are becoming important targets for cancer therapy, and inhibitors targeting Wnt, Notch, and Hedgehog have reached clinical evaluation (see footnote 3). The low expression of DKK3 in several human cancers combined with its cancer specific apoptosis inducing effects suggest that re-establishing DKK3 holds promise as a novel targeted cancer therapy. The use of demethylating agents to re-express *DKK3* in cancers where promoter hypermethylation leads to gene silencing have been shown in studies using cell lines of several cancers including gastric, prostate, lung, and leukemia (reviewed by Veeck and Dahl, 2012). Demethylating agents azacitidine and decitabine have been approved in the treatment of myelodysplastic syndromes (Cataldo et al., 2009; Santos et al., 2010), however this method is not gene specific and could alter the epigenetic patterns of the entire genome. In neuroblastoma, demethylating agents are unlikely to be of therapeutic use, as low DKK3 expression was not shown to be due to promoter methylation (Haug et al., 2011). An alternative strategy involves DKK3 targeted gene therapy. The vast majority of work to date has been carried out Ad-REIC/DKK3 (Abarzua et al., 2005), however the use of a biodegradable cationic polymer based vector has also been reported (Veeck and Dahl, 2012). Ad-REIC/DKK3 has been used with success in preclinical studies demonstrating robust anti-tumor effects including induction of cancer cell specific apoptosis and inhibition of metastatic disease (Abarzua et al., 2005; Edamura et al., 2007; Kawasaki et al., 2009; Sakaguchi et al., 2009; Than et al., 2011), and is currently in clinical trials for use in prostate cancer (see footnote 3; NCT01197209). In addition, DKK3 also appears to play a role in chemoresistance, and Ad-REIC/DKK3 mediated downregulation of MDR-1 expression through JNK activation sensitized previously chemoresistant tumor cells to chemotherapy, which supports the applicability of using Ad-REIC/DKK3 in combination with convention chemotherapies in the treatment of drug-resistant cancers (Kawasaki et al., 2009; Hirata et al., 2012). Preclinical studies to evaluate the effect of Ad-REIC/DKK3 in neuroblastoma are yet to be conducted.

Alternative strategies more specific to neuroblastoma may involve the direct targeting of the oncogenic *miR-17-92* cluster to re-establish *DKK3* expression in *MYCN* amplified neuroblastoma, or the use of agents which directly affect *MYCN* expression in neuroblastoma cells such as the BET inhibitor, JQ1 (Mertz et al., 2011). Certainly, targeting miRNAs as a new class of therapeutics has been gaining increased interest in recent years, and Miravirsen, which inhibits *miR-122* is currently in phase II trials for the treatment of patients with Hepatitis C virus (Hu et al., 2012).

## Conclusions and Perspectives

In recent years, the involvement of the p53/MDM2/p14^ARF^ pathway in neuroblastoma has become increasingly evident and targeting this pathway through small molecule inhibitors of the MDM2-p53 interaction in neuroblastoma which remains predominantly wt even at relapse, is an attractive therapeutic option. Currently newer more potent MDM2-p53 antagonists are undergoing commercial development, which will be tested in suitable preclinical models of neuroblastoma leading to their evaluation in early phase clinical trials and hopefully eventually into standard neuroblastoma therapy.

The precise involvement of SKP2 and DKK3 in neuroblastoma will require further investigation and these targets are currently much less developed in terms of their therapeutic potential. Nevertheless their involvement in other cancers as well as neuroblastoma makes them a priority for commercial and academic drug development programs.

It is envisaged that the therapeutic strategies which have been proposed in this review are most likely to be effective in combination with existing treatment regimens or other targeted agents and not alone. The increased understanding of neuroblastoma tumorigenesis and mechanisms of therapy resistance and relapse, advances in the development of murine models which more closely resemble high-risk metastatic and/or relapsed disease, and the drive towards targeted/personalized therapies, will hopefully provide increased opportunities to test out some of the paradigms mentioned in this review. When testing therapies directed against all these targets it will be important to do so both in *MYCN* amplified and non-amplified settings to fully evaluate their dependence on MYCN.

This is an exciting time for neuroblastoma drug development and with many new targeted agents in the pipeline undergoing preclinical evaluation and early phase clinical trials it surely will not be long before one reaches frontline therapy and improves the outcome, whilst hopefully reducing long-term toxicity in patients with high-risk disease.

## Conflict of Interest Statement

The authors declare that the research was conducted in the absence of any commercial or financial relationships that could be construed as a potential conflict of interest.

## Footnotes

^1^http://www-p53.iarc.fr/

^2^http://p53.free.fr/

^3^http://www.clinicaltrials.gov

^4^http://www.ptcbio.com/ptc596
